# Thiophene- and Carbazole-Substituted *N*-Methyl-Fulleropyrrolidine Acceptors in PffBT4T-2OD Based Solar Cells

**DOI:** 10.3390/ma13061267

**Published:** 2020-03-11

**Authors:** Hugo Gaspar, Flávio Figueira, Karol Strutyński, Manuel Melle-Franco, Dzmitry Ivanou, João P. C. Tomé, Carlos M. Pereira, Luiz Pereira, Adélio Mendes, Júlio C. Viana, Gabriel Bernardo

**Affiliations:** 1 IPC/i3N—Institute for Polymers and Composites, University of Minho, Campus de Azurém, 4800–058 Guimarães, Portugal; hugo.da.silva.gaspar@gmail.com (H.G.); jcv@dep.uminho.pt (J.C.V.); 2 QOPNA & LAQV-REQUIMTE, Department of Chemistry, University of Aveiro, 3810–193 Aveiro, Portugal; jtome@tecnico.ulisboa.pt; 3 CICECO—Aveiro Institute of Materials, Department of Chemistry, University of Aveiro, 3810–193 Aveiro, Portugal; strutynski.karol@gmail.com (K.S.); manuelmelle.research@gmail.com (M.M.-F.); 4 LEPABE, Department of Chemical Engineering, University of Porto, 4200–465 Porto, Portugal; ivanou@fe.up.pt (D.I.); mendes@fe.up.pt (A.M.); 5 CQE, Departamento de Engenharia Química, Instituto Superior Técnico, Universidade de Lisboa, Av. Rovisco Pais, n1, 1049–001 Lisboa, Portugal; 6 Department of Chemistry, University of Porto, Rua do Campo Alegre, s/n, 4169–007 Porto, Portugal; cmpereir@fc.up.pt; 7 Department of Physics and i3N—Institute for Nanostructures, Nanomodelling and Nanofabrication, University of Aveiro, 3810–193 Aveiro, Portugal; luiz@ua.pt

**Keywords:** polymer solar cells, fulleropyrrolidine acceptors, C_70_ mono-adducts, regioisomers, stereoisomers

## Abstract

The impact of fullerene side chain functionalization with thiophene and carbazole groups on the device properties of bulk-heterojunction polymer:fullerene solar cells is discussed through a systematic investigation of material blends consisting of the conjugated polymer poly[(5,6-difluoro-2,1,3-benzothiadiazol-4,7-diyl)-alt-(3,3‴-di(2-octyldodecyl)-2,2′;5′,2″;5″,2‴-quaterthiophen-5,5‴-diyl)] (PffBT4T-2OD) as donor and C_60_ or C_70_ fulleropyrrolidines as acceptors. The photovoltaic performance clearly depended on the molecular structure of the fulleropyrrolidine substituents although no direct correlation with the surface morphology of the photoactive layer, as determined by atomic force microscopy, could be established. Although some fulleropyrrolidines possess favorable lowest unoccupied molecular orbital levels, when compared to the standard PC_71_BM, they originated OPV cells with inferior efficiencies than PC_71_BM-based reference cells. Fulleropyrrolidines based on C_60_ produced, in general, better devices than those based on C_70_, and we attribute this observation to the detrimental effect of the structural and energetic disorder that is present in the regioisomer mixtures of C_70_-based fullerenes, but absent in the C_60_-based fullerenes. These results provide new additional knowledge on the effect of the fullerene functionalization on the efficiency of organic solar cells.

## 1. Introduction

Organic photovoltaic (OPV) cells are a promising solar energy harvesting technology because of their flexibility, light weight, and compatibility with large-scale production using roll-to-roll methods (R2R) that are expected to reduce the module fabrication cost and the energy payback time [1,2,3]. Much effort has been made in the last two decades—focusing in the material design, device engineering, and morphology optimization—aiming to increase the power conversion efficiencies (*PCE*) of the OPV device. In the last few years, OPVs have developed significantly, attaining recently *PCE* values > 16% for single junction devices [4,5,6] and > 17% for tandem cells [7].

The active layer in a OPV device is made of a blend of a p-type polymer and an n-type acceptor forming a bicontinuous interpenetrating network, known as bulk-heterojunction (BHJ). The p-type small band gap polymer poly[(5,6-difluoro-2,1,3-benzothiadiazol-4,7-diyl)-alt-(3,3‴-di(2-octyldodecyl)-2,2′;5′,2″;5″,2‴-quaterthiophen-5,5‴-diyl)], known as PffBT4T-2OD, or as PCE11, has recently become popular in the OPV field [8,9,10,11,12,13,14,15] due to its high hole mobility of 1.5–3.0 × 10^−2^ cm^2^V^−1^s^−1^ [10], associated with its high crystallinity, that allows its use in relatively thick (~300 nm) and high efficiency solar cells. The n-type acceptors used in OPVs can be either fullerene derivatives or non-fullerene small molecules. Although the performance of OPVs using non-fullerene acceptors has already outperformed its fullerene-based counterpart, the research in polymer:fullerene solar cells using new ‘non-standard’ fullerenes remains very active [16,17,18,19,20,21,22].

Buckminsterfullerene C_60_ was the first fullerene used in an OPV device, in the seminal work by Sariciftci et al. [23] reporting the discovery of the photo-induced electron-transfer from a conducting polymer to C_60_. However, the very low solubility of C_60_ in common organic solvents makes it very difficult to process and therefore, soon after its introduction in the OPV field, the strategy of functionalizing C_60_ with solubilizing moieties was adopted. For this reason, the fullerenes (6,6)-Phenyl-C61-butyric acid methyl ester PC_61_BM [24,25] and its analogue (6,6)-Phenyl-C71-butyric acid methyl ester PC_71_BM [26] soon emerged as the two most widely used electron accepting materials in OPVs. These two fullerenes, PC_61_BM and PC_71_BM, are now utilized as reference acceptors for all kinds of other fullerene acceptors, because of their good solubility, high electron mobility, and high chemical stability. A key difference between PC_61_BM and PC_71_BM is the broader photo-absorption profile of PC_71_BM in the visible region of the solar spectrum that allows increased photon harvesting and a potentially higher photocurrent for devices using PC_71_BM rather than PC_61_BM, an important attribute that has brought the C_70_ analogue to the forefront of OPV research (despite its higher cost) [27,28].

Fullerenes and derivatives display several physical and chemical properties that make them appealing for use in OPVs and other applications [29,30,31]. Fullerenes with proper organic moieties can be tuned on their solubility, highest occupied and lowest unoccupied molecular orbital (HOMO/LUMO) levels, orientation, and crystallinity in the solid state and on their surface energy, among others [32]. Several different fullerene functionalization strategies have been previously tested aiming to improve the performance of polymer:fullerene solar cells [16,17,33]. Some important examples of alternative fullerene acceptor families include dihydronaphthyl-based fullerenes [34,35,36,37] and fulleropyrrolidines [38,39,40,41,42,43,44,45,46,47].

Fulleropyrrolidines have several attractive features namely: they can be synthesized from pristine C_60_ and C_70_ in a simple one-step synthetic procedure using Prato reaction conditions [48]; ease of modification of the substituents bonded to the pyrrolidine ring by using as reactants commercially available or readily prepared glycine and aldehyde derivatives, and a good chemical stability. One of the first reports on the use of fulleropyrrolidine acceptors in OPVs, was made by Lee et al. [38] in 2007. These authors synthesized fulleropyrrolidine derivatives substituted with different chain lengths and tested them in OPVs with standard geometry and using the polymer MEH-PPV. The efficiencies obtained were all very low (≤0.47%) and no comparison was made with reference devices using PC_61_BM. Matsumoto et al. [39] synthesized and tested a series of fulleropyrrolidine derivatives in P3HT-based OPV cells with standard geometry. Several of the fulleropyrrolidine derivatives originated devices that outperformed reference devices with PC_61_BM. The best fulleropyrrolidine-based devices attained a *PCE* of 3.44% compared to 2.53% for PC_61_BM-based reference devices. A new phenothiazine-containing fulleropyrrolidine derivative was synthesized and tested by Mi et al. [40] in P3HT-based devices with standard geometry. However, the devices achieved the best modest efficiency of only 1.42%. Saravanan et al. [41] synthesized two fullerene-terthiophene dyads without (3T-C_60_) and with (3TH-C_60_) hexyl chains on the terthiophene substituent and tested them in P3HT devices with standard geometry. The 3TH-C60 fullerenes originated better devices (max *PCE* of 2.54%) than 3T-C60 due to their higher miscibility with the P3HT matrix. Zhang et al. [42] synthesized two indole-containing fullerene derivatives and tested them in inverted solar cells with the structure of ITO/ZnO/P3HT:fullerene/MoO_3_/Ag. The best devices showed a *PCE* of 3.32%, very similar to the *PCE* of 3.28% for similar reference PC_61_BM based devices. Six novel soluble (60)fulleropyrrolidine derivatives were synthesized by Karakawa et al. [43] and tested in OPVs with P3HT and with PTB7. Devices with P3HT were made with the standard geometry ITO/PEDOT:PSS/BHJ/Al and devices with PTB7 were made with inverted geometry ITO/PFN/BHJ/MoOx/Al. The best P3HT-based devices using fulleropyrrolidine derivatives achieved a max. *PCE* of 2.41% comparable to the value of 2.30% obtained for reference P3HT devices with PC_61_BM. Interestingly, the authors also observed that although the lowest unoccupied molecular orbital (LUMO) levels of all the fulleropyrrolidines closely resemble each other and those of PC_61_BM, devices with P3HT displayed a large variety of *PCE*s ranging from 0.02% to 2.41% depending on the acceptor. In the case of PTB7-based devices using fulleropyrrolidines a max *PCE* of 7.34% was achieved, slightly above the value of 7.03% obtained for reference PTB7 devices with PC_61_BM. Kaunisto et al. [44] synthesized eight fulleropyrrolidine derivatives with thiophene substituents ranging from 1 to 4 thiophene units and tested them in inverted BHJs using P3HT as p-type material. Fulleropyrrolidine derivatives with one or two thiophene units performed better as acceptor materials than those with three or four thiophene units. Interestingly, even though all the devices with different fulleropyrrolidine derivatives exhibited very similar BHJ surface morphologies, as determined by atomic force microscopy (AFM), and all the fulleropyrrolidine derivatives exhibited very similar LUMO levels (either −3.7 eV or −3.6 eV), as determined by cyclic voltammetry, the produced devices exhibited largely different *PCE* ranging from <0.01% to 2.0%, and all of them inferior to the *PCE* of reference P3HT:PC_61_BM devices. Pitliya et al. [45] synthesized a novel fulleropyrrolidine derivative C_60_-fused *N*-(3methoxypropyl)-2-(carboxyethyl)-5-(4-cyanophenyl)fulleropyrrolidine (NCPF) and tested it in P3HT-based OPV devices with standard geometry ITO/PEDOT:PSS/BHJ/Al. Fulleropyrrolidine-based devices achieved a *PCE* of 1.77% comparable to 2.14% for reference devices based on PC_61_BM. Eight fulleropyrrolidine derivatives with alternating *N*-phenyl or *N*-methyl group were synthesized by Liang et al. [46] and tested in P3HT-based OPVs with standard geometry ITO/PEDOT:PSS/BHJ/Ca/Al. The devices produced using different fulleropyrrolidine derivatives exhibited very different photovoltaic properties with *PCE*s ranging from 0.70% to 3.19%, compared with the *PCE* value of 3.32% for reference devices with PC_61_BM. Interestingly, even though some of the fullerenes exhibited more favorable LUMO levels than PC_61_BM, they originated devices with considerably lower *V_OC_* and *PCE* values. Yamane et al. [47] synthesized two fulleropyrrolidine derivatives with the benzophenone moiety that can supress their crystallization and tested them in P3HT-based OPV devices with structure ITO/MoO_3_/BHJ/Al. The devices exhibited higher thermal stability but lower *PCE* than reference devices with PC_61_BM.

In this work, we synthesize eight novel thiophene- and carbazole-substituted N-methyl-fulleropyrrolidine acceptors and test their impact on the performance of polymer solar cells based on the polymer PffBT4T-2OD. Although the final figures of merit are lower compared to similar OPVs with standard PC_71_BM, the knowledge acquired on the influence of fulleropyrrolidine functionalization on the final device performances can open a new framework for the synthesis and use of such acceptors.

## 2. Materials and Methods

### 2.1. Materials

The pristine fullerenes C_60_ (˃99.5% purity) with *M_w_* = 720.64 g.mol^−1^ and C_70_ (˃99% purity) with *M_w_* = 840.77 g.mol^−1^, used in the synthesis of novel fullerene derivatives were purchased from Solenne BV, as well as the PC_61_BM (*M_w_* = 910.88 g·mol^−1^, 99% purity) used in cyclic voltammetry measurements.

The following materials, purchased from Ossila Ltd., were used in device fabrication: (i) Poly(3,4-ethylenedioxy-thiophene):poly(styrene sulfonic acid) (PEDOT:PSS, Heraeus Clevios AI4083); (ii) the polymer PffBT4T-2OD with *M_n_* = 83,008 g.mol^−1^ and *M_w_* = 172,033 g.mol^−1^ (catalogue # M302); and (iii) the reference fullerene PC_71_BM (M113), with empirical formula C_82_H_14_O_2_ and *M_w_* = 1030.99 g mol^−1^. The high purity grade solvent *o*-dichlorobenzene (*o*-DCB), purchased from Sigma-Aldrich, was used in device fabrication. All these materials and solvent were used as received without further purification.

### 2.2. NMR Spectroscopy

Bruker Avance 300 or 500 (300 or 500.13 MHz for ^1^H and 125.76 MHz for ^13^C) spectrometers (Billerica, Massachusetts, EUA) were used to record the ^1^H and ^13^C solution NMR spectra of the functionalized fullerenes. The solvents used were CS_2_, deuterated acetone and deuterated chloroform (99.6%, TCI Chemicals) and the internal reference was tetramethylsilane (TMS). The chemical shifts are expressed in (ppm).

### 2.3. Preparation of Compounds **60A**–**60D** and **70A**–**70D**

A solution containing 100 mg (0.14 mmol) of C_60_ or C_70_, *N*-methylglycine (0.35 mmol) and of the corresponding aldehyde (0.67 mmol) was stirred until reflux temperature. After this 0.67 mmol of the corresponding aldehyde was added to the reaction every 3 h (3 times) and the reaction was maintained for 24 h, then the solvent was removed in vacuum. The solid residue was purified by flash column chromatography (eluent: toluene/hexanes 1:3 with increasing amounts of toluene until purification of the first brown band) affording roughly 30-40% of the *N*-methyl-3,4-fulleropyrrolidine derivatives **60D** and **70D**. NMR spectra and characterization are shown in Supplementary Information (Figures S1–S24).

### 2.4. Cyclic Voltammetry

The electrochemical experiments were performed using an Autolab PGSTAT302N potentiostat. A three-electrode cell arrangement was used to record the voltammograms; a polished glassy-carbon (GC) pin (3 mm in diameter) was used as a working electrode, a platinum wire as a counter electrode, and a nonaqueous Ag|Ag^+^ reference electrode with an internal solution of AgNO_3_ (0.01 M) and 0.1 M of Bu_4_NPF_6_ in acetonitrile. The fullerenes (ca. 0.6 mg/mL) were dissolved in a solvent mixture of 4:1 (by volume) chlorobenzene (CB): acetonitrile and 0.1 M Bu_4_NPF_6_ was added as a supporting electrolyte. Before measurements, the solutions were deaerated by means of a 7 min purge with high purity Argon. A potential scan rate of 100 mV/s was used to record the cyclic voltammograms. The Argon flow was kept above the solution in the cell, throughout the measurements. All electrode potentials were quoted with respect to equilibrium potential (*E*_1/2_) of Fc+/Fc redox couple in the same solvent mixture; *E*_1/2_ (Fc^+^/Fc) = 0.29 V vs. Ag|Ag^+^. The LUMO and HOMO energy levels were estimated from the onset potential of the reduction ($E_{Red}^{on}$) and oxidation ($E_{Ox}^{on}$) respectively: *E_LUMO_* = −4.9 − $E_{Red}^{on}$; *E_HOMO_* = −4.9 − $E_{Ox}^{on}$.

### 2.5. Ab Initio DFT Calculations

We performed density functional theory calculations at the PBEh-3c level to compute all molecular structures [49]. The electronic structure energies reported were computed at the PBE-def2-TZVP level which has been found to show better accuracy than hybrid functionals for these systems [50,51,52]. For C_70_ systems all isomers and diestereoisomers—α1, α2, β1, and β2—were explicitly computed. The ORCA 4.2 program was used to perform all the calculations [53].

### 2.6. Absorption Spectroscopy

Initially, the optical properties of the fulleropyrrolidines were characterized, in *o*-DCB solution, by means of UV–vis absorption spectroscopy. Due to the difficulty in preparing homogeneous spin-coated thin films of the fullerenes on quartz windows, we were unable to measure the UV–vis spectra of these. UV–vis absorption spectroscopy was later used to evaluate the effect of the prepared fulleropyrrolidines in the light absorption profiles of the different polymer:fullerene blends. Absorption spectra (UV–vis), of the blend thin solid-state films deposited in quartz substrates, were obtained on a Shimadzu UV-2501PC spectrophotometer, in the 350–800 nm range.

### 2.7. Device Fabrication

OPV devices were made with a standard structure ITO/PEDOT:PSS/Active layer/Ca/Al. ITO (Indium Tin Oxide) has a sheet resistance of 20 Ω/☐. Blend solutions of the polymer PffBT4T-2OD and the fullerene derivatives, with concentrations of respectively 4 mg·mL^−1^ and 4.8 mg·mL^−1^ (1:1.2 mass ratio) were prepared and then spin-coated on top of ITO substrates pre-coated with PEDOT:PSS. Other PffBT4T-2OD:fullerene mass ratios were also tested initially (1:3 and 3:1) but they produced lower device efficiencies. Contrary to previous work with PffBT4T-2OD using solvent mixtures of CB:*o*-DCB (1:1) to dissolve the polymer, here it proved necessary to use a pure *o*-DCB solvent (which has a stronger solvent power than the standard CB:*o*-DCB mixture) due to the high *M_w_* of the polymer used. Similarly to previous reports on fulleropyrrolidine based OPVs [42], no additives were used because preliminary tests have shown that they did not promote any improvements in efficiency. The blend solutions were pre-heated to 120 °C and then they were spin-coated, in a nitrogen filled glove box and at a spin speed of 800 rpm, onto PEDOT:PSS/glass substrates that had been pre-heated to 120 °C. The as-deposited active layer films were then left drying inside the glove box for 1 h. Finally, the top electrode—composed of 5 nm calcium and 100 nm aluminum—was evaporated sequentially on top of the active layer, under a pressure < 2 × 10^−6^ mbar.

### 2.8. Device Performance Characterization

A Newport-Oriel 96000 AM 1.5 Global solar simulator, which had been calibrated using an NREL standard silicon solar cell to ensure an irradiance level of 1000 W/m^2^, was used to measure the photovoltaic properties of the devices. An aperture mask (E521 from Ossila Ltd.) was used to limit the light-exposed area of the device to 2.6 mm^2^. All measurements were performed at room temperature.

### 2.9. Atomic Force Microscopy (AFM)

The surface morphology of the PffBT4T-2OD: fullerene thin films was imaged using atomic force microscopy (AFM) in tapping mode. AFM measurements were performed using a Molecular Imaging PicoLE AFM in contact mode and several scans were imaged in flattened mode data to remove the background slope. The scan size of topographic images was 5 × 5 μm in all experiments.

## 3. Results and Discussion

**Figure 1:**
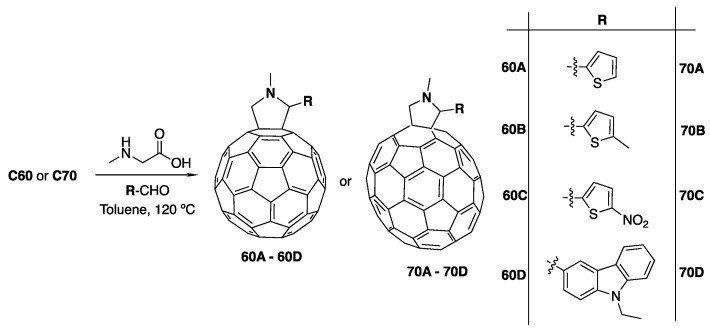
Synthetic method used in the preparation of compounds **60A** to **60D** and **70A** to **70D**.

Several C_60_ and C_70_ fulleropyrrolidines were synthesized following the Prato methodology [48] where the appropriate azomethine ylide precursors are generated using *N*-methyl glycine (sarcosine) and the corresponding aldehyde, in this case thiophene and carbazole moieties (Figure 1).

These precursors react readily with C_60_ and C_70_ providing fulleropyrrolidines containing substituted thiophene and carbazole moieties. Herein, this was achieved by a controlled cycloaddition reaction in which a pyrrolidine ring is fused with a (6,6) ring junction of both C_60_ or C_70_.

Fulleropyrrolidines **60A** to **60D** were synthesized using the 1,3-dipolar cycloaddition of the corresponding thiophene and carbazole aldehydes and C_60_, with *N*-methylglycine and paraformaldehyde in toluene under reflux. After purification by column chromatography on silica gel compounds **60A** to **60D** were obtained in around 40% yield. **70A** to **70D** were prepared in the same manner replacing C_60_ with C_70_. The final structures were confirmed by ^1^H NMR with spectroscopy assignments supported by 2D-H experiments.

It is possible to note that the solubility of the resulting C_70_ derivatives was improved when compared with the solubility of the substituted C_60_. This became particularly evident when preparing concentrated fullerene solutions (~20 mg·mL^−1^) for ^13^C NMR analysis—while all the C_70_ derivatives were soluble or partially soluble in deuterated chloroform CDCl_3_ (C_70_ bearing the carbazole unit showed to be a little less soluble), the C_60_ derivatives were not soluble in CDCl_3_, and could be completely solubilized only in CS_2_ (the most powerful solvent for fullerenes). This higher solubility of C_70_ derivatives in organic solvents, compared to the corresponding C_60_ derivatives, has been previously reported [54,55]. The ^1^H NMR spectra of the C_60_ derivatives are quite simple and shows the characteristic features of a *N*-methyl-(60)fulleropyrrolidine mono-adducts. The ^1^H NMR spectra of the C_70_ derivatives exhibited four singlets and four sets of doublets (*J* ≈ 9.5 Hz) for the pyrrolidine protons, demonstrating the presence of four different isomeric products of C_70_ mono-adducts (α1, α2, β1, and β2, as represented in Figure S25). Unlike C_60_, in which all carbon atoms and double bonds are initially equivalent, the lower symmetry of C_70_ gives rise to an array of different reactivities. Integration of spectra based on the pyrrolidine protons allowed us to estimate the fractions of each isomer (Table 1).

**Figure 2:**
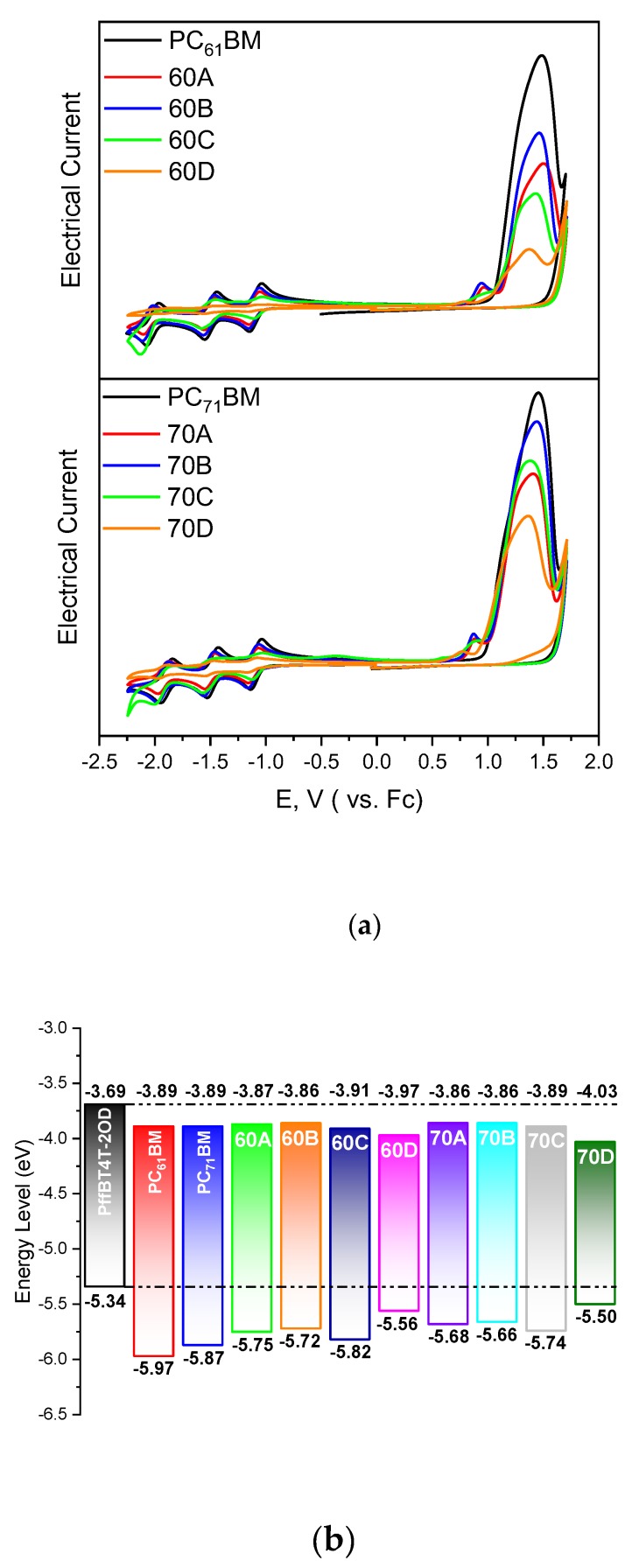
(**a**) Cyclic voltammetry results for all different fullerenes. The electrical current scale is shifted arbitrarily for clarity. (**b**) HOMO and LUMO levels for all materials as calculated from cyclic voltammetry. The HOMO and LUMO levels for PffBT4T-2OD, as indicated in literature [10], are also shown.

The cyclic voltammograms of the eight synthesized fulleropyrrolidine acceptors, as well as the voltammograms of the corresponding PC_61_BM and PC_71_BM references are shown in Figure 2a. The corresponding HOMO/LUMO levels determined from these voltammograms are shown in Table S1 in Supplementary Information and represented in Figure 2b. Also represented in Figure 2b are the HOMO/LUMO levels of the polymer, from the literature [10]. The HOMO/LUMO levels that we have determined for PC_61_BM and PC_71_BM are in excellent agreement with literature [56,57] which attests the reliability of our measurements for the fulleropyrrolidine acceptors. The fulleropyrrolidines **60A**, **60B**, **70A**, and **70B** have slightly higher LUMO levels than the PC_71_BM standard which should favor higher *V_OC_* and *PCE* values assuming that all other factors remain unchanged.

**Figure 3:**
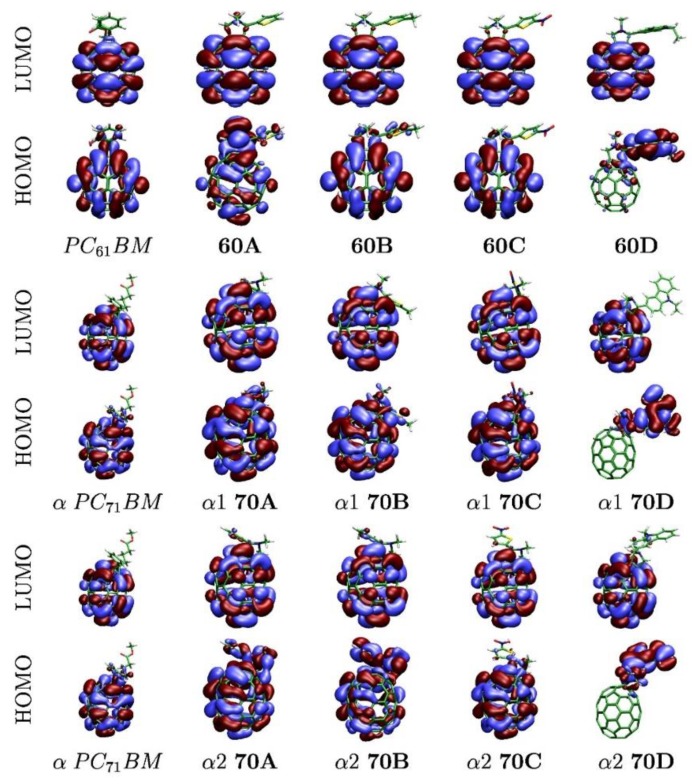

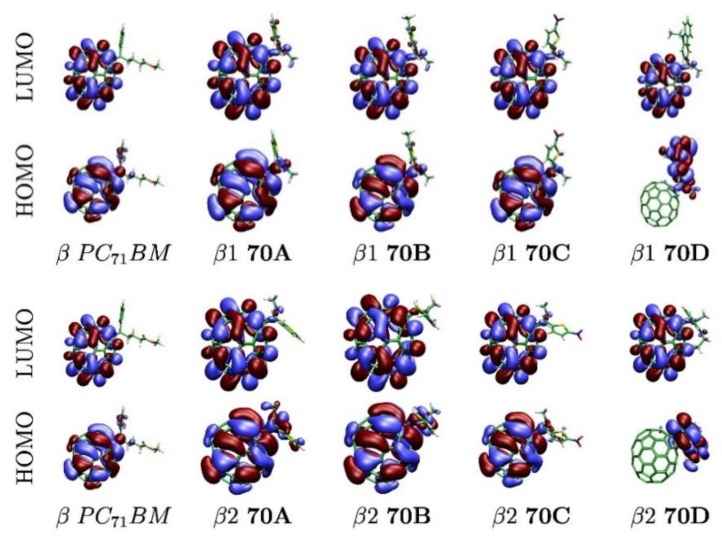
Frontier orbitals of pristine and functionalized C_60_ and C_70_ molecules.

The DFT computed HOMO and LUMO energies show a moderate agreement with the experimental values, Table 2. The LUMO energies show the best agreement, with differences up to 0.17 and 0.28 eV for **70C** and **70D** respectively. For C_70_, α1 and α2 diastereoisomers generally yield very similar frontier orbital energies with the exception of α1-**70D** and α2-**70D** HOMOs which differ by 0.08 eV. Also, the β1 and β2 HOMO and LUMO energies are very similar. Remarkably, when α and β C_70_ isomers are systematically compared, HOMOs show lower energies, while LUMOs show higher energies. Thus, all α isomers have larger HOMO-LUMO gaps than β isomers. Figure 3 shows the electron density for both frontier orbitals for all molecules. The HOMO orbitals are localized on the carbazole moiety for **60D** and **70D** structures. In all the other cases, the HOMO electron sit predominantly on the fullerene part, although for some systems—namely **60A**, α-**70A**, α-**70B**, and β2-**70B**—some density is also found on the functional groups. Interestingly, the LUMO orbitals, associated with the capacity of these molecules to act as electron acceptors, reside on the fullerene for all structures.

The UV–vis absorption spectra recorded between 300 and 800 nm for the four C_60_ derivatives and the four C_70_ derivatives in *o*-DCB are shown in Figure S26. The C_70_ derivatives have a larger photo-absorption profile in the visible region. The absorption bands of the C_60_ derivatives are similar and the same happens with the absorption bands of the C_70_ derivatives. In agreement with the literature [43,46,47], the C_60_ derivatives display a large peak at ~330 nm, a small peak at ~430 nm and a very weak absorption peak ~700 nm.

**Figure 4:**
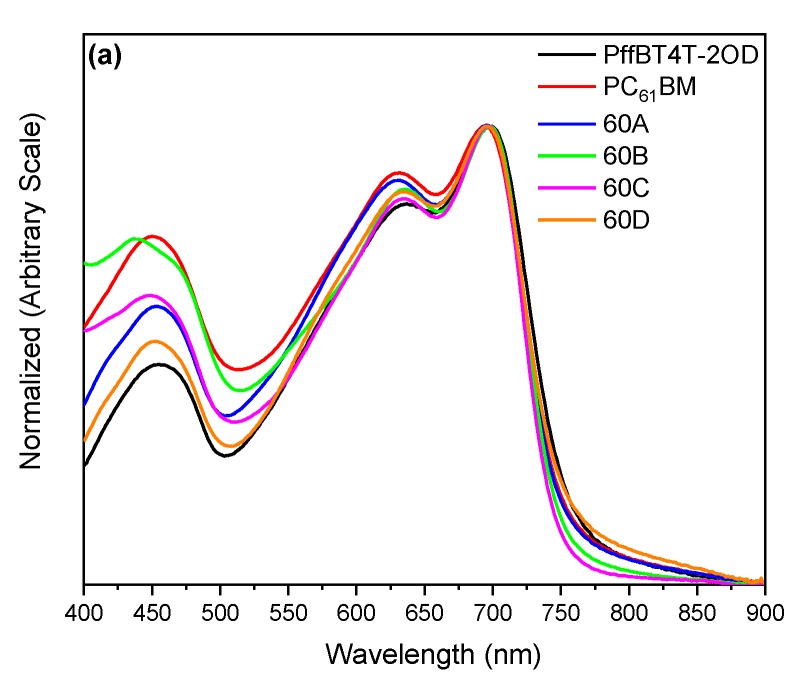

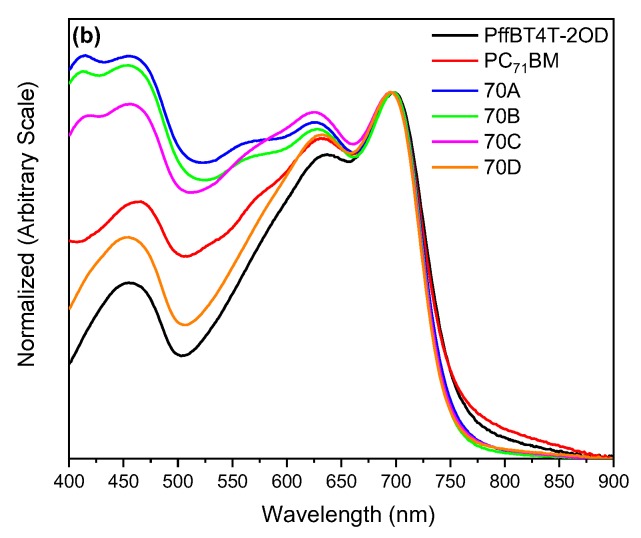
UV–vis spectra of a pristine PffBT4T-2OD film and of PffBT4T-2OD:fullerene blend films with: (**a**) C_60_-fullerenes and (**b**) C_70_-fullerenes. All spectra are normalized to the intensity of the polymer 0-1 transition peak at ~700 nm.

Figure 4 shows the UV–vis spectra of the blends and pure polymer, normalized to the intensity of their 0–1 transition peak at ~700 nm. As expected, considering the absorption spectra of the individual fullerenes shown in Figure S26, the BHJs with C_70_-fulleropyrrolidines exhibit a stronger light absorption in the range 400–700 nm than the corresponding BHJs with C_60_-fulleropyrrolidines.

**Figure 5:**
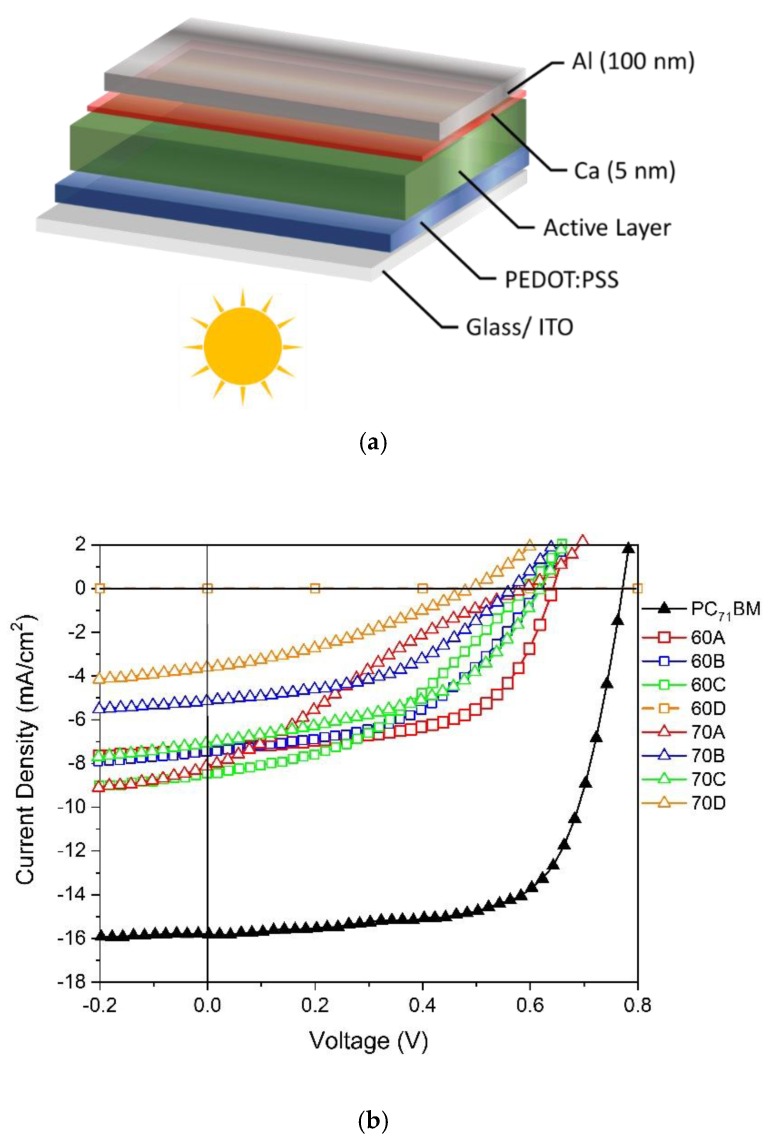

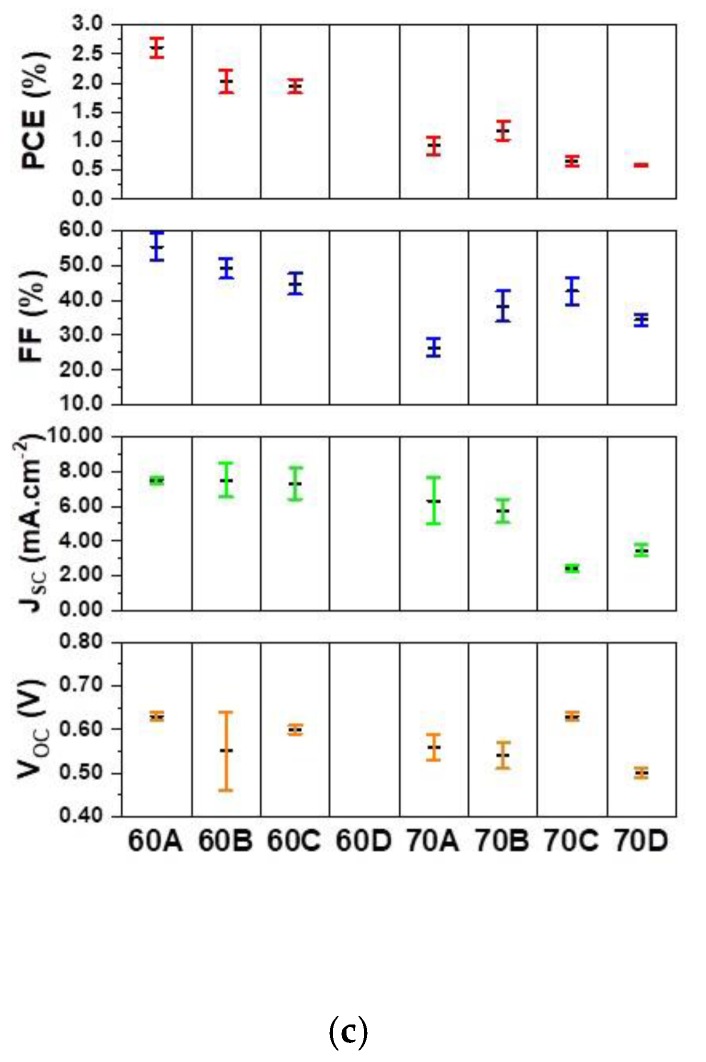
(**a**) A schematic of the standard structure of the devices; (**b**) Representative J–V curves for PffBT4T-2OD based devices with the PC_71_BM standard and with the eight fulleropyrrolidine acceptors 60A–60D and 70A–70D; (**c**) Overall device metrics for the eight different PffBT4T-2OD:fulleropyrrolidine based devices.

Figure 5a represents the standard architecture ITO/PEDOT:PSS/BHJ/Ca/Al of the devices considered in the present work. The current density–voltage (J–V) curves of devices processed with the eight fulleropyrrolidines, as well as a PC_71_BM-based control device, are shown in Figure 5b. The corresponding figures of merit are shown in Figure 5c and in Table 3.

As it is evident from Figure 5b,c and Table 3, all the fulleropyrrolidines originated devices with photovoltaic performances lower than reference devices based on PC_71_BM. All the J–V curves of devices with fulleropyrrolidine acceptors show some space charge limited current (SCLC) effects, and interestingly devices based on 70A display a characteristic S-shaped similar to S-shaped J–V curves that have been previously reported in fulleropyrrolidine-based OPVs [43]. We note that previous work has often reported novel fullerenes that despite displaying more favorable LUMO levels than PC_61_BM and PC_71_BM, gave rise to OPV devices with considerable lower *PCE* [34,46,47,58,59,60,61,62,63,64] and also with simultaneously lower *PCE* and *V_OC_* [34,46,47,62,63,64] than reference devices based on PC_61_BM and PC_71_BM. In the present work, among our fulleropyrrolidine-based devices the best efficiencies (η) are achieved by devices with the C_60_ derivatives **60A** (2.78%), **60B** (2.20%), and **60C** (2.04%). These results show that a higher efficiency is obtained when the thiophene moiety does not contain any substituent in the remaining alpha position and the efficiency tends to decrease when this position is substituted with electron withdrawing substituents. It is also evident from Figure 5c and Table 3 that, in general, the C_60_ derivatives originate higher performing devices than the C_70_ derivatives, even though the latter have broader light absorption in the visible region as depicted in Figure 4 and Figure S26. The only exception occurs in compounds 60D and 70D, where 60D originates no efficiency and 70D originates an efficiency of 0.60%. However, this exception is most likely associated with the solubility differences observed between 60D (low solubility) and 70D (more soluble) as the use of derivatives with a higher solubility tends to facilitate the process of thin film formation and dispersion in the BHJ, therefore increasing the efficiency of the devices. C_70_-based devices exhibit in general lower efficiency than C_60_-based devices, and in our opinion this is most probably due to the structural disorder (as shown in Table 1) and to the energetic disorder (as shown in Table 2) introduced in these systems by the presence of several C_70_-fulleropyrrolidine isomers. In fact, as our DFT calculations (Table 2) show, different isomers of C_70_-fulleropyrrolidine have systematically different electronic properties. To further support these ideas, we note that for example, according to Umeyama et al. [65], in the particular case of the PC_71_BM mixture of isomers, the β1-isomer present in the mixture of four isomers (α1, α2, β1, and β2) does not contribute to the efficiency of the mixture.

**Figure 6:**
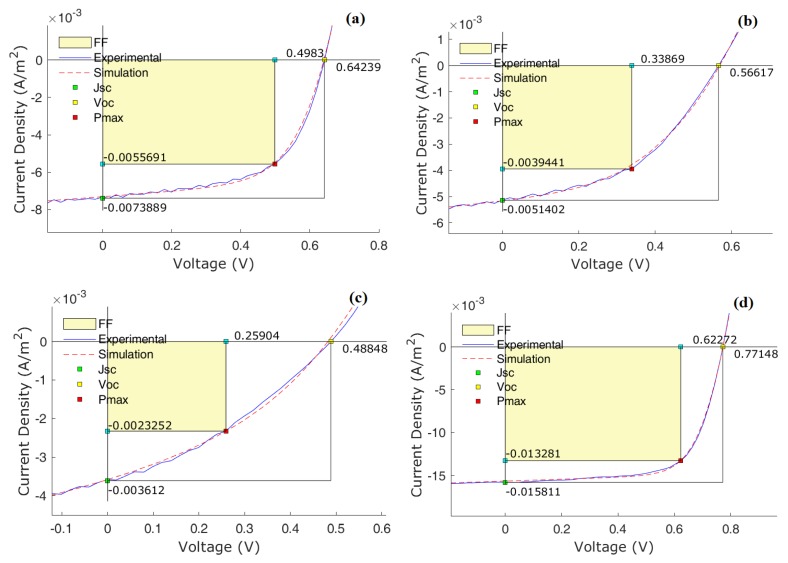
Experimental (solid lines) and simulated (dashed lines) J–V curves for OPVs based in donors (**a**) **60A**, (**b**) **60C**, (**c**) **70C**, and (**d**) PC_71_BM.

The experimental J–V data was fitted, using genetic algorithms, to the OPV equivalent circuit [66]. The most relevant simulated J–V curves are displayed in Figure 6 and the full simulated data is presented in Table 4, namely the simulated values of *J_ph_*, R_s_ and R_p_. Analysis of our data shows that there is no obvious relationship between the *V_OC_* values obtained and the $E_{LUMO}^{A}-E_{HOMO}^{D}$ energy level differences, as determined by cyclic voltammetry, although for samples 60A and 60B some trend was observed (as previous indicated and expected). This means that locally defined energy levels, arising from structural defects and/or inhomogeneous film formation, need to be taken into consideration. In fact, as previous work has shown, energetic disorder at D:A interphase can contribute for changing significantly the *V_OC_* values and this should be also occurring in the present work. The equivalent circuit R_p_ (modelling physical electrical carrier’s loss) helps, in some way, to support this hypothesis: higher R_p_ corresponds in general to effectively higher η. Moreover, and besides these factors influencing *V_OC_*, a decrease of R_p_ usually leads to a decrease of *V_OC_* (equivalent circuit) and in general this behavior is observed, namely with samples 60A, 60B, and 60C and with samples 70B, 70C, and 70D. However, we must take into account that we are comparing different samples (structure, LUMO/HOMO levels, and solubility) and therefore specific considerations for each of them should be also considered.

The series resistance R_s_—considering the usual high fluctuation of this parameter—exhibits for the C_60_ series an expected relationship with η. An opposite relationship between R_s_ and R_p_ is observed in our samples, for C_60_ series (except 60D that we will discuss later): increasing the first (higher electrode/bulk resistance), the other decreases (higher electrical loss) which results in a loss of efficiency. However, it is interesting to note that *J_SC_* increases regardless the increase of R_s_ and the decrease of R_p_ (samples 60A, 60B, and 60C) suggesting a clear issue with the BHJ morphology and phase separation. On the other hand, for the C_70_ samples two situations are of special interest: samples 70A and 70C. For the former, a high R_s_ together with very low R_p_ suggests an I–V behavior departing from the typical electrical circuit model and this is confirmed by the strong SCLC influence in the J–V curve (although a surprisingly high *J_SC_*) that markedly decreases the fill factor (FF). For the later and despite the moderately high R_p_ and low R_s_ values, the results are far from what we would a priori expect, even more having a favorable LUMO level. Clearly, a low *J_SC_* is a problem and considering that the light absorption of the blend with this donor displays an average behavior, we conclude that the energetic disorder should have a relevant influence in the OPVs made with this fullerene.

As a final evidence supporting the impact of the BHJ morphology on the macroscopic device behavior, we can observe that, as all the figures of merit of the OPVs degrade, the J–V curve clearly becomes an overlap of a diode characteristic curve with the expected curve from a charge transport under space charge (SCLC) conditions. Moreover, a simple comparison between *J_SC_* and *J_ph_* (estimated from the simulations and corresponding to the pure generated electrical carriers—power source in the equivalent electrical circuit) shows noticeable loss during the transport process. According to this, a degradation of the ideal equivalent electrical circuit behavior, should imply a decrease of the FF (observed in general) with further loss of efficiency. As all the active layers were deposited following the same procedure, it can be assumed that the different fulleropyrrolidine acceptors originate morphologically different films with significant influence in the electrical charge transport.

Notwithstanding the very low efficiency of the 60D-based devices, the discussion of their device metrics is interesting. In fact, OPVs made with the fulleropyrrolidine 60D have nearly zero efficiency (<0.1%) mainly due to a nearly zero *J_SC_*. Even though such extremely low *PCE* values, a suitable fit to the electrical equivalent circuit could be performed, showing that a very high R_s_ is present (over 6 KΩ). Nevertheless, an optimal high value of R_p_ is achieved, indicating that the loss in electrical carrier transport is low. Moreover, a moderate FF is obtained. However, the high *V_OC_* is typical of a ‘single layer’ OPV, i.e., just one material (donor or acceptor) is contributing to the electrical charge generation and transport. Considering that 70D-based devices also exhibit the worst efficiency among the C_70_ derivatives, it is clear that the carbazole substituent must originate a marked morphology degradation resulting in poorer D:A interfaces. This is most likely due to the lower solubility that the carbazole moiety imparts to the corresponding fulleropyrrolidine in organic solvents, and which should also reflect itself in a much less favorable polymer–fullerene interaction [67,68].

**Figure 7:**
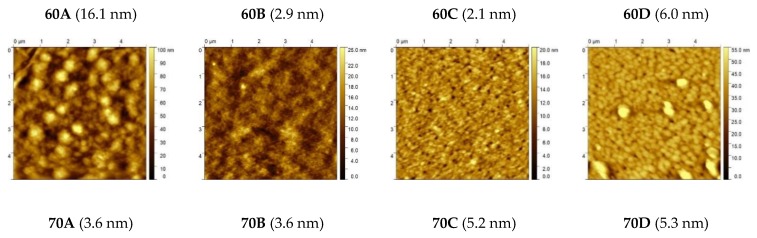

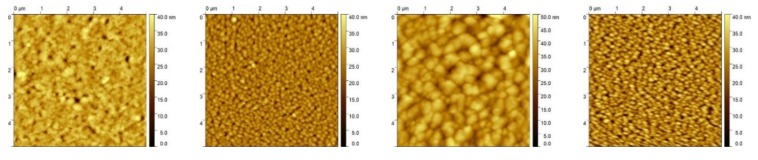
Morphological AFM images of BHJ films with different acceptors.

We have performed AFM analysis, in tapping mode, of the surface morphology of the different BHJ films, and representative height images are shown in Figure 7, together with the associated values of root mean square (*rms*) roughness inside brackets. The corresponding phase images are shown in Figure S27. However, when trying to correlate the surface morphologies with the device efficiencies the results are inconclusive as no clear correlation between the film morphologies and the OPV performances can be observed. For example, even though films 60A and 60C present respectively the higher (16.1 nm) and lower (2.1 nm) values of *rms* roughness among all the eight devices, their corresponding device *PCE*s are very similar (maximum values of 2.78% and 2.04%, respectively) and both are considerably higher than any of the 70A–70D based devices. These observations are not surprising considering that AFM only analyses the surface morphology of the films and this can be very different from the bulk morphology. A detailed morphological characterization of these films would require, besides the measurement of the size of phase domains, the measurement of the degree of purity and the degree of orientation (crystallinity) inside those phase domains and this would only be possible through the use of some hardly accessible techniques such as small angle neutron scattering (SANS) [11,69], neutron reflectivity [70], resonant soft X-ray scattering (RSoXS) [71], and grazing-incidence wide-angle X-ray scattering (GIWAXS) [72] and was beyond the scope of this work.

We note that the fullerene functionalization besides changing the HOMO/LUMO levels also affects several other properties with direct impact on the corresponding device efficiency. Different functionalization of the C_60_ and C_70_ cages, can modify dramatically their solid-state packing and crystallization properties and the electronic coupling between adjacent fullerenes, causing a significant change in their local electron mobility [73]. Furthermore, they can also modify the compatibility between the polymer and the fullerene and as a result the level of fullerene dispersion in the polymer matrix [67,68,74]. Consequently, several other parameters can be affected including the electron mobility of the fullerene phase in the BHJ [19,75] and the LUMO level of the fullerene. Regarding this latter issue, for example Durrant et al. [76] have shown that PC_61_BM aggregation in the BHJ lowers systematically its LUMO level and variations in the film morphology can change the device V_OC_ by up ∼0.2 V [77]. Piersimoni et al. [78] also demonstrated that fullerene crystallization reduces the energy of the charge transfer state (*E*_CT_) and this causes a reduction in *V_OC_*.

Finally, we refer to a 2017 study, by Karakawa et al. [79] of OPV devices using PEDOT: PSS as hole transport layer (HTL) and an active layer with *N*-alkyl-fulleropyrrolidine acceptors. These authors have identified an interfacial reaction between the basic *N*-alkyl-fulleropyrrolidine acceptors and the acidic PEDOT:PSS that affects the OPV performance. Interestingly, fulleropyrrolidines have been investigated as electron acceptors in OPVs for more than a decade, and although several works have published device data on inverted devices without PEDOT:PSS, the reason for that choice of device architecture was never mentioned before the work by Karakawa et al. This is an interesting topic that we will address with great detail in a future publication, by testing these same fullerenes in PEDOT:PSS-free devices with inverted architectures. For now, however, we believe from our data that the electrical transport, due to non-ideal D:A interphase separation, should be the main explanation for the obtained results.

## 4. Conclusions

We have demonstrated a simple approach for the chemical modification of N-methyl-fulleropyrrolidine acceptors that can be extended to thiophene and carbazole based moieties. However, OPV devices based on these novel fullerene acceptors performed worse than PC_71_BM-based reference devices. Despite these lower performances obtained, our results confirm the critical dependence of the figures of merit of devices on the chemical structures of the fullerene derivative acceptors. As a main conclusion, it seems to be clear that the structural and energetic heterogeneity present in the isomer mixture of C_70_-fulleropyrrolidines is detrimental to device performance and this emphasizes the need to develop regioselective synthetic pathways.

## Figures and Tables

**Table 1:** Estimation in percentage of the fraction of isomers present in C_70_ derivatives.

	α1 (%)	α2 (%)	β1(%)	β2 (%)
**70A**	30	20	25	24
**70B**	30	27	25	17
**70C**	47	18	20	18
**70D**	*	*	*	*

* NMR is too complex to make a correct estimation

**Table 2:** Experimental and computed HOMO and LUMO energies at the PBE-def2-TZVP/PBEh-3c level. All values in eV.

	HOMO					LUMO				
	Exp.	α1	α2	β1	β2	Exp.	α1	α2	β1	β2
**C_60_**		−5.80					−4.10			
**C_70_**		−5.80					−4.03			
**PC_61_BM**	−5.97	−5.47				−3.89	−3.94			
**PC_71_BM**	−5.87	−5.52		−5.44		−3.89	−3.85		−3.87	
**60A**	−5.75	−5.40				-3.87	−3.90			
**60B**	−5.72	−5.36				−3.86	−3.88			
**60C**	−5.82	−5.60				−3.91	−4.06			
**60D**	−5.56	−5.09				−3.97	−3.84			
**70A**	−5.68	−5.47	−5.47	−5.40	−5.38	−3.86	−3.79	−3.79	−3.84	−3.83
**70B**	−5.66	−5.43	−5.42	−5.37	−5.35	−3.86	−3.77	−3.77	−3.82	−3.83
**70C**	−5.74	−5.69	−5.69	−5.61	−5.60	−3.89	−4.01	−4.00	-4.06	−4.04
**70D**	−5.50	−5.06	−5.05	−5.04	−5.12	−4.03	−3.76	−3.75	−3.80	−3.79

**Table 3:** Device metrics for cells with different acceptors, showing the peak and (average of 8 devices) *PCE* values.

PffBT4T-2OD	*PCE* (%)	V_OC_ (V)	FF (%)	J_sc_ (mA/cm^2^)
**PC_71_BM**	8.41 (8.19 ± 0.24)	0.72 (0.74 ± 0.02)	71.2 (69.8 ± 1.8)	16.41 (15.87 ± 0.40)
**60A**	2.78 (2.61 ± 0.16)	0.64 (0.63 ± 0.01)	58.7 (55.3 ± 3.9)	7.36 (7.48 ± 0.15)
**60B**	2.20 (2.02 ± 0.20)	0.62 (0.55 ± 0.09)	47.9 (49.2 ± 2.9)	7.46 (7.51 ± 0.98)
**60C**	2.04 (1.94 ± 0.12)	0.59 (0.60 ± 0.01)	40.7 (44.8 ± 3.0)	8.46 (7.30 ± 0.90)
**60D**	(0.07)	(1.09)	(45.6)	(0.15)
**70A**	1.15 (0.92 ± 0.15)	0.59 (0.56 ± 0.03)	24.2 (26.4 ± 2.5)	8.08 (6.32 ± 1.30)
**70B**	1.33 (1.18 ± 0.16)	0.57 (0.54 ± 0.03)	45.9 (38.4 ± 4.4)	5.14 (5.74 ± 0.68)
**70C**	0.75 (0.65 ± 0.08)	0.62 (0.63 ± 0.01)	47.5 (42.6 ± 4.0)	2.52 (2.43 ± 0.16)
**70D**	0.60 (0.59 ± 0.02)	0.49 (0.50 ± 0.01)	34.2 (34.4 ± 1.6)	3.61 (3.46 ± 0.34)

**Table 4:** Generated photocurrent (*J_ph_*) and parallel (R_p_) and series resistances (R_s_) determined by fitting the equivalent circuit to the experimental data.

	*J_ph_* (mA/cm^2^)	R_s_ (Ω)	R_p_ (Ω)
**PC_71_BM**	16.41	159	9.72 × 10^4^
60A	7.36	225	3.43 × 10^4^
60B	7.65	633	2.40 × 10^4^
60C	8.86	1157	1.39 × 10^4^
60D	(0.15)	(6032)	(9.72 × 10^4^)
70A	8.57	1721	0.57 × 10^4^
70B	5.33	1235	2.14 × 10^4^
70C	2.53	619	2.99 × 10^4^
70D	3.80	1644	1.41 × 10^4^

## Supplementary Materials

The following are available online at https://www.mdpi.com/1996-1944/13/6/1267/s1, Table S1. HOMO and LUMO levels for all materials as calculated from cyclic voltammetry; Figure S1 to S24: ^1^H NMR, ^13^C NMR, and HSQC spectra of derivatives **60A** to **60D** and **70A** to **70D**; Figure S25. Structure of the four different C_70_-fulleropyrrolidine isomers; Figure S26. UV–vis spectroscopy of the fullerenes in 1,2-dichlorobenzene (o-DCB); Figure S27. AFM phase images.
