# Supplementary material for: Thiophene- and Carbazole-Substituted N-Methyl-Fulleropyrrolidine Acceptors in PffBT4T-2OD Based Solar Cells
Source: Materials (Basel). 2020 Mar 11;13(6):1267. doi: 10.3390/ma13061267 (PMC7142714; doi:10.3390/ma13061267)
Supplement: Supplementary file 1 [file materials-13-01267-s001.pdf]

materials

MDPI

Article

Thiophene- and Carbazole-Substituted *N*-Methyl-Fulleropyrrolidine Acceptors in PffBT4T-2OD Based Solar Cells

Hugo Gaspar [1], Flávio Figueira [2,3,*], Karol Strutyński [3], Manuel Melle-Franco [3], Dzmitry Ivanou [4], João P. C. Tomé [2,5], Carlos M. Pereira [6], Luiz Pereira [7], Adélio Mendes [4], Júlio C. Viana [1], and Gabriel Bernardo [4,*]

[1] IPC/i3N—Institute for Polymers and Composites, University of Minho, Campus de Azurém, 4800–058 Guimarães, Portugal; hugo.da.silva.gaspar@gmail.com (H.G.); jcv@dep.uminho.pt (J.C.V.)
[2] QOPNA & LAQV-REQUIMTE, Department of Chemistry, University of Aveiro, 3810–193 Aveiro, Portugal; jtome@tecnico.ulisboa.pt
[3] CICECO—Aveiro Institute of Materials, Department of Chemistry, University of Aveiro, 3810–193 Aveiro, Portugal; strutynski.karol@gmail.com (K.S.); manuelmelle.research@gmail.com (M.M.-F.)
[4] LEPABE, Department of Chemical Engineering, University of Porto, 4200–465 Porto, Portugal; ivanou@fe.up.pt (D.I.); mendes@fe.up.pt (A.M.)
[5] CQE, Departamento de Engenharia Química, Instituto Superior Técnico, Universidade de Lisboa, Av. Rovisco Pais, n1, 1049–001 Lisboa, Portugal; jtome@tecnico.ulisboa.pt
[6] Department of Chemistry, University of Porto, Rua do Campo Alegre, s/n, 4169–007 Porto, Portugal; cmpereir@fc.up.pt
[7] Department of Physics and i3N—Institute for Nanostructures, Nanomodelling and Nanofabrication, University of Aveiro, 3810–193 Aveiro, Portugal; luiz@ua.pt
* Correspondence: gbernardo@fe.up.pt (G.B.) and ffigueira@ua.pt (F.F.)

Received: 7 February 2020; Accepted: 9 March 2020; Published: date

Table S1. HOMO and LUMO levels for all materials as calculated from cyclic voltammetry. The potential onsets used for the calculations are also indicated.

Material	E_{ox}^{onset} (V)	HOMO (eV)	E_{red}^{onset} (V)	LUMO (eV)
**PffBT4T-2OD**	-	-5.34	-	-3.69
**$PC_{61}BM$**	1.07	-5.97	-1.01	-3.89
**$PC_{71}BM$**	0.97	-5.87	-1.01	-3.89
**60A**	0.85	-5.75	-1.03	-3.87
**60B**	0.82	-5.72	-1.04	-3.86
**60C**	0.92	-5.82	-0.99	-3.91
**60D**	0.66	-5.56	-0.93	-3.97
**70A**	0.78	-5.68	-1.04	-3.86
**70B**	0.76	-5.66	-1.04	-3.86
**70C**	0.84	-5.74	-1.01	-3.89
**70D**	0.60	-5.50	-0.87	-4.03

NMR Characterization

60A: ^{1}H NMR (300 MHz, CS2 + Acetone-d6) δ 7.41 (d, *J* = 4.903 Hz, 2H), 7.06 (dd, *J* = 4.801, 3.819 Hz, 1H), 5.33 (s, 1H), 5.01 (d, *J* = 9.574 Hz, 1H), 4.32 (d, *J* = 9.534 Hz, 1H), 2.93 (s, 3H); ^{13}C NMR (126 MHz, Acetone-d6) δ 156.83, 154.67, 154.10, 153.91, 147.83, 147.56, 147.03, 146.87, 146.83, 146.76, 146.72, 146.65, 146.62, 146.48, 146.36, 146.15, 146.12, 145.92, 145.84, 145.82, 145.79, 145.68, 145.29, 145.24, 144.95, 143.70, 143.55, 143.25, 143.15, 142.85, 142.82, 142.75, 142.70, 142.63, 142.61, 142.60, 142.50,

142.43, 142.24, 142.18, 141.32, 140.73, 140.70, 140.45, 140.14, 137.62, 137.24, 136.55, 136.30, 128.60, 127.47, 127.24, 79.52, 77.77, 70.35, 69.34, 40.57. (Figures S1, S2 and S3, below)

60B: ^{1}H NMR (500 MHz, CS2 + Acetone-d6) δ 7.22 (d, *J* = 3.5 Hz, 1H), 6.80 – 6.63 (m, 1H), 5.27 (s, 1H), 5.02 (d, *J* = 9.5 Hz, 1H), 4.31 (d, *J* = 9.6 Hz, 1H), 2.93 (s, 3H), 2.55 (s, 3H); ^{13}C NMR (126 MHz, CS2 + Acetone-d6) δ 156.86, 154.73, 154.24, 154.08, 147.80, 147.61, 147.21, 147.02, 146.85, 146.80, 146.73, 146.69, 146.62, 146.59, 146.45, 146.36, 146.11, 146.09, 145.89, 145.84, 145.78, 145.66, 145.26, 145.23, 144.92, 143.67, 143.52, 143.22, 143.14, 143.12, 142.83, 142.79, 142.73, 142.69, 142.60, 142.52, 142.44, 142.23, 142.18, 141.52, 140.69, 140.45, 140.20, 138.77, 137.60, 137.25, 136.44, 136.27, 128.75, 125.55, 79.84, 77.72, 70.24, 69.31, 40.54. (Figures S4, S5 and S6, below)

60C: ^{1}H NMR (300 MHz, CS2 + Acetone-d6) δ 7.84 (dd, *J* = 4.3, 1.2 Hz, 1H), 7.43 (dd, *J* = 4.3, 1.1 Hz, 1H), 5.35 (d, *J* = 2.3 Hz, 1H), 5.05 (d, *J* = 9.8 Hz, 1H), 4.37 (dd, *J* = 9.8, 1.0 Hz, 1H), 3.01 (d, *J* = 1.0 Hz, 3H); ^{13}C NMR (75 MHz, Acetone-d6) δ 150.53, 146.99, 143.28, 129.61, 128.88, 128.88, 128.29, 125.97, 117.32, 109.61, 79.11, 70.38, 40.67. (Figures S7, S8 and S9, below)

60D: ^{1}H NMR (300 MHz, CS2 + Chloroform) δ 7.47 – 7.26 (m, 3H), 7.19 – 7.03 (m, 3H), 5.20 – 4.91 (m, 2H), 4.44 – 4.24 (m, 3H), 2.83 (s, 3H), 1.46 (t, *J* = 7.1 Hz, 3H). (Figures S10, S11 and S12, below)

70A: ^{1}H NMR (300 MHz, Chloroform-d) δ 7.8 – 7.3 (m, 2H), 7.2 – 6.8 (m, 3H), 4.8 – 4.1 (m, 3H), 3.7 – 2.9 (m, 3H), 2.8 – 2.4 (m, 3H); ^{13}C NMR (126 MHz, Chloroform-d) δ 151.83, 151.37, 151.05, 150.77, 150.66, 150.34, 149.84, 149.41, 149.34, 149.09, 147.47, 147.42, 147.11, 147.06, 146.94, 146.18, 145.91, 145.44, 145.28, 143.33, 142.92, 140.48, 137.31, 133.86, 132.37, 131.66, 131.34, 131.25, 39.82, 34.15, 31.96, 26.75, 25.36, 22.73, 21.35, 14.16. (Figures S13, S14 and S15, below)

70B: ^{1}H NMR (300 MHz, Chloroform-d) δ 7.02 – 6.42 (m, 1H), 4.82 – 3.97 (m, 2H), 3.79 – 3.03 (m, 1H), 2.75 – 2.24 (m, 6H); ^{13}C NMR (75 MHz, Chloroform-d) δ 165.39, 163.79, 160.74, 160.72, 159.36, 158.10, 156.70, 155.19, 151.75, 150.64, 149.30, 149.28, 146.95, 146.92, 144.62, 143.28, 143.21, 138.35, 137.33, 135.35, 133.84, 131.22, 130.11, 128.23, 126.90, 125.69, 124.42, 64.12, 60.09, 39.76, 29.72, 28.04, 15.81, 15.55. (Figures S16, S17 and S18, below)

70C: ^{1}H NMR (500 MHz, Chloroform-d) δ 8.19 – 7.42 (m, 1H), 7.19 – 6.99 (m, 1H), 4.83 – 4.41 (m, 1H), 4.34 – 3.99 (m, 1H), 3.67 – 3.14 (m, 1H), 2.73 – 2.43 (m, 3H); ^{13}C NMR (126 MHz, Chloroform-d) δ 157.01, 156.20, 154.26, 151.64, 151.48, 151.27, 150.86, 150.79, 150.74, 150.01, 149.92, 149.43, 149.27, 149.14, 148.97, 148.78, 148.06, 147.56, 147.21, 147.07, 146.09, 145.96, 143.58, 143.48, 143.39, 143.06, 140.63, 133.99, 133.70, 133.31, 131.81, 131.64, 131.36, 128.66, 127.94, 127.56, 127.32, 127.10, 126.79, 75.71, 70.60, 70.32, 68.93, 66.28, 61.90, 60.29, 53.59, 40.04. (Figures S19, S20 and S21, below)

70D: ^{1}H NMR (500 MHz, CS2 + Acetone-d6) δ 8.72 – 6.98 (m, 7H), 4.71 – 4.18 (m, 2H), 3.61 (dd, *J* = 35.4, 7.7 Hz, 1H), 2.56 (d, *J* = 11.3 Hz, 6H), 1.50 – 1.09 (m, 5H), 1.03 – 0.78 (m, 3H); ^{13}C NMR (126 MHz, CS2 + Acetone-d6) δ 151.64, 151.54, 151.49, 151.44, 151.30, 151.28, 151.20, 151.16, 151.09, 150.87, 150.84, 150.75, 150.71, 150.65, 150.60, 150.54, 150.48, 150.34, 150.31, 150.14, 149.99, 149.94, 149.90, 149.85, 149.81, 149.70, 149.57, 149.51, 149.47, 149.45, 149.36, 149.30, 149.27, 149.21, 149.16, 149.09, 149.07, 149.01, 148.94, 148.84, 148.75, 148.68, 148.47, 148.37, 148.23, 148.09, 148.05, 148.00, 147.96, 147.75, 147.60, 147.56, 147.47, 147.41, 147.33, 147.20, 147.15, 147.09, 147.07, 146.97, 146.90, 146.87, 146.86, 146.81, 146.67, 146.62, 146.57, 146.38, 146.35, 146.23, 146.12, 145.92, 145.79, 145.75, 145.73, 145.68, 145.52, 145.32, 145.19, 145.00, 144.94, 144.86, 144.72, 144.65, 144.42, 144.20, 143.91, 143.85, 143.71, 143.58, 143.50, 143.41, 143.35, 143.27, 143.22, 143.18, 143.12, 142.92, 142.86, 142.67, 142.40, 142.21, 141.92, 141.80, 141.69, 141.22, 140.98, 140.92, 140.78, 140.55, 140.42, 140.36, 140.18, 140.01, 139.88, 139.74, 137.98, 137.84, 137.58, 137.30, 133.89, 133.65, 133.58, 133.14, 132.79, 132.36, 132.29, 132.11, 132.04, 131.73, 131.69, 131.58, 131.48, 131.30, 131.25, 131.20, 131.13, 130.82, 130.64, 129.12, 128.40, 128.03, 127.28, 126.95, 126.84, 126.74, 126.32, 126.18, 125.82, 123.11, 122.78, 120.95, 120.70, 120.66, 119.62, 119.52, 119.48, 119.44, 108.89, 108.79, 108.74, 108.70, 84.00, 83.15, 80.97, 73.36, 71.56, 70.59, 70.32, 69.72, 68.77, 68.24, 67.18, 66.86, 66.36, 62.12, 60.39, 58.81, 58.42, 39.97, 39.64, 39.59, 39.56, 38.02, 37.92, 37.82, 37.78, 29.61, 29.46, 29.30. (Figures S22, S23 and S24, below)

NMR Spectra

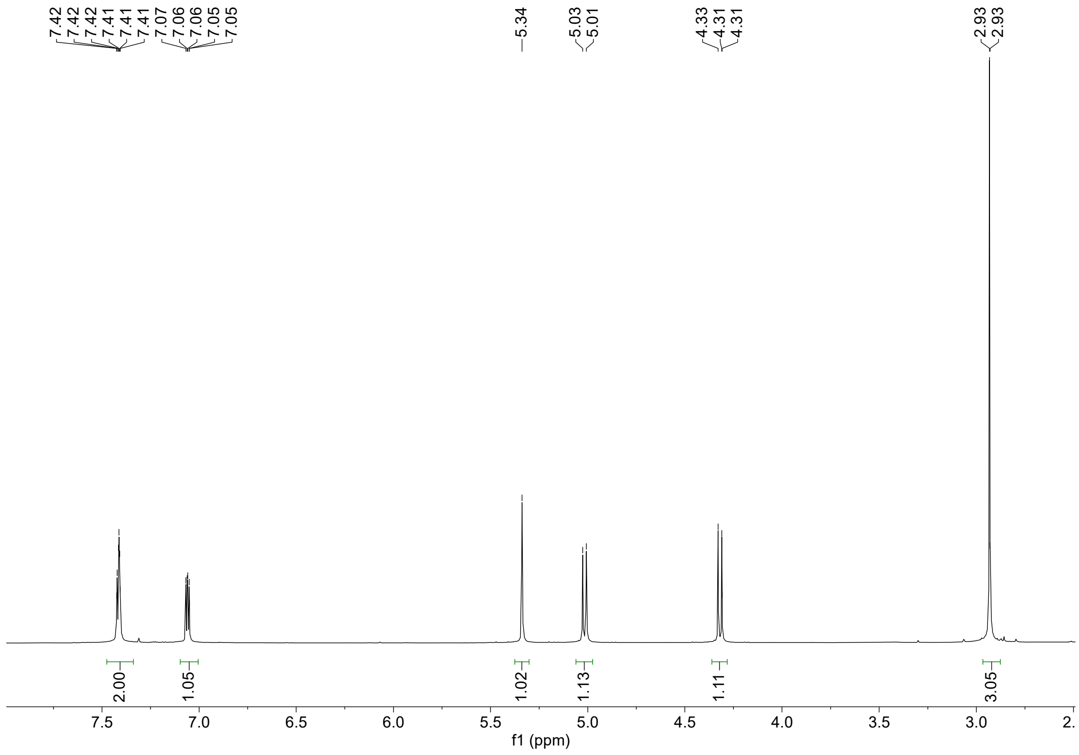

Figure S1. ^{1}H NMR spectrum of compound 60A in a mixture of CS2 and acetone-d6.

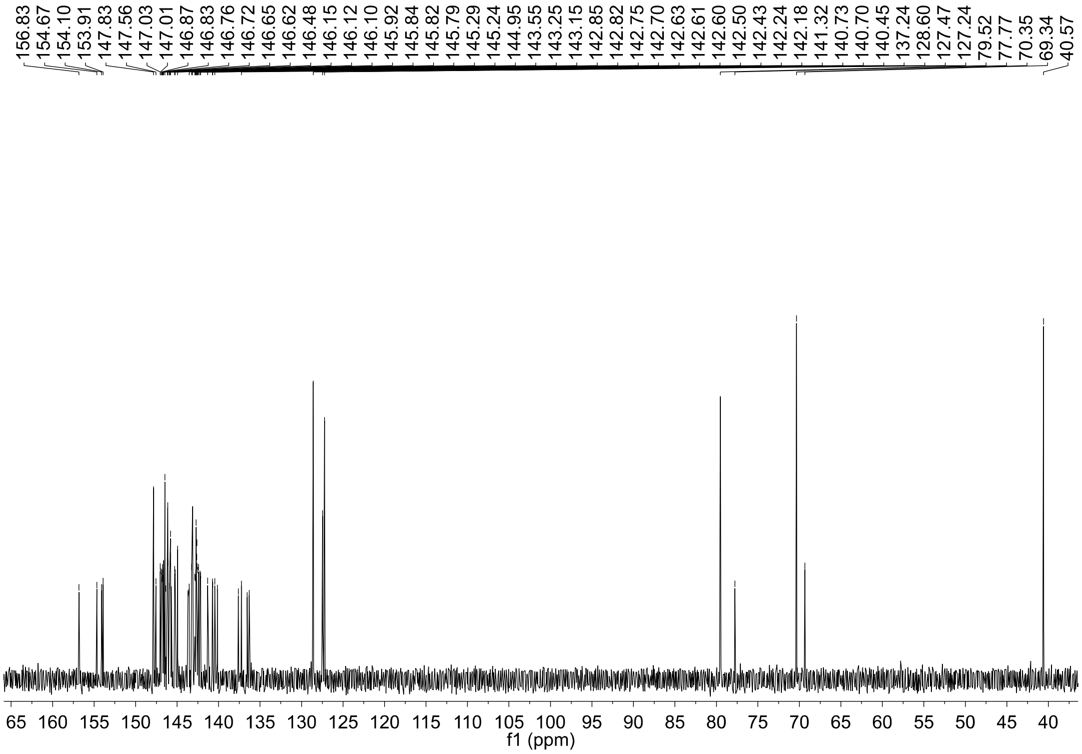

Figure S2. ^{13}C NMR spectrum of compound 60A in a mixture of CS_2 and acetone-d6.

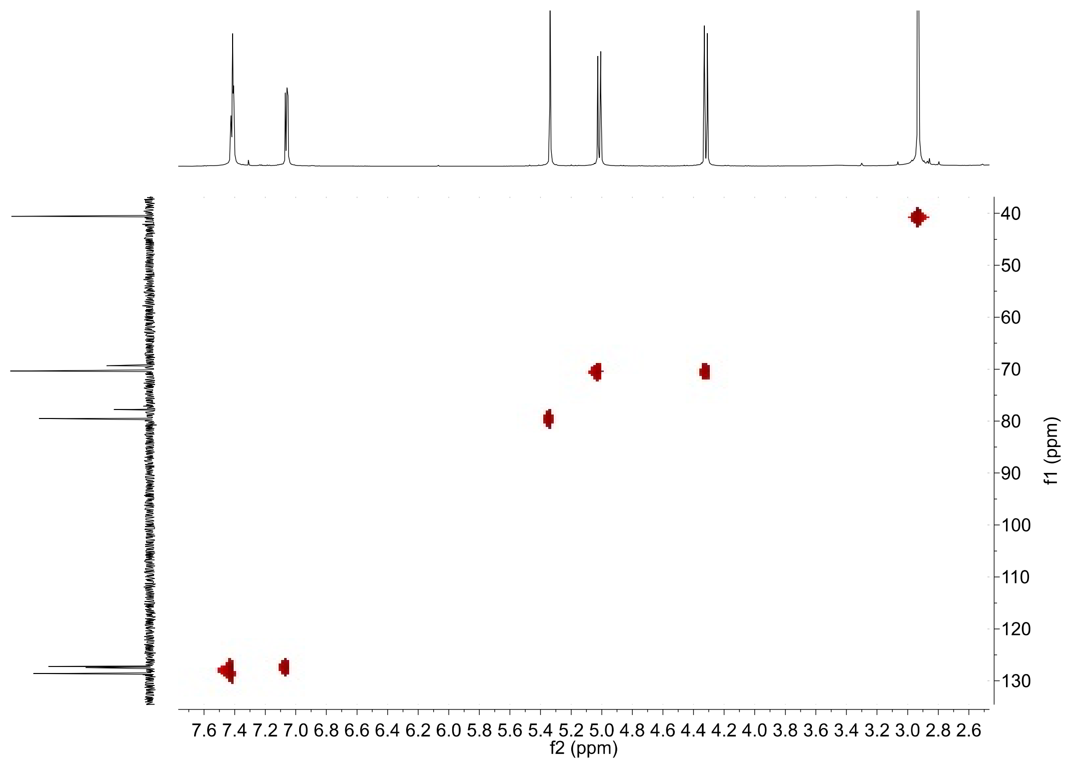

Figure S3. HSQC spectrum of compound 60A in a mixture of CS_2 and acetone-d6.

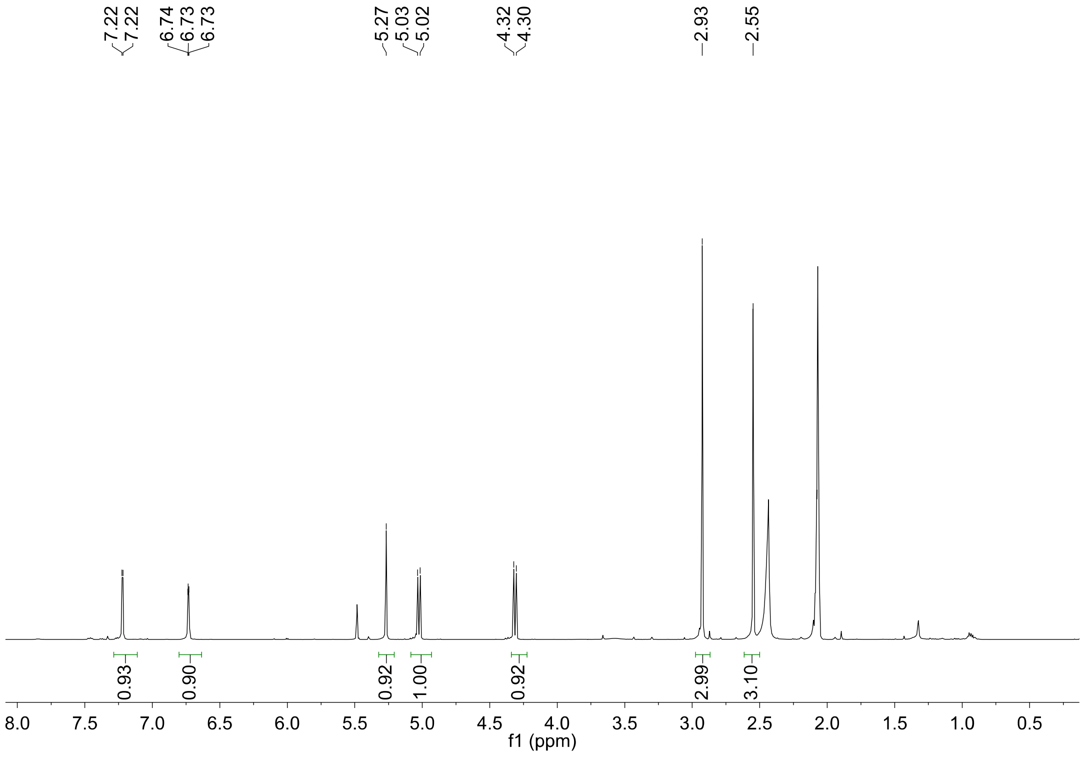

Figure S4. ^1H NMR spectrum of compound 60B in a mixture of CS_2 and acetone-d6.

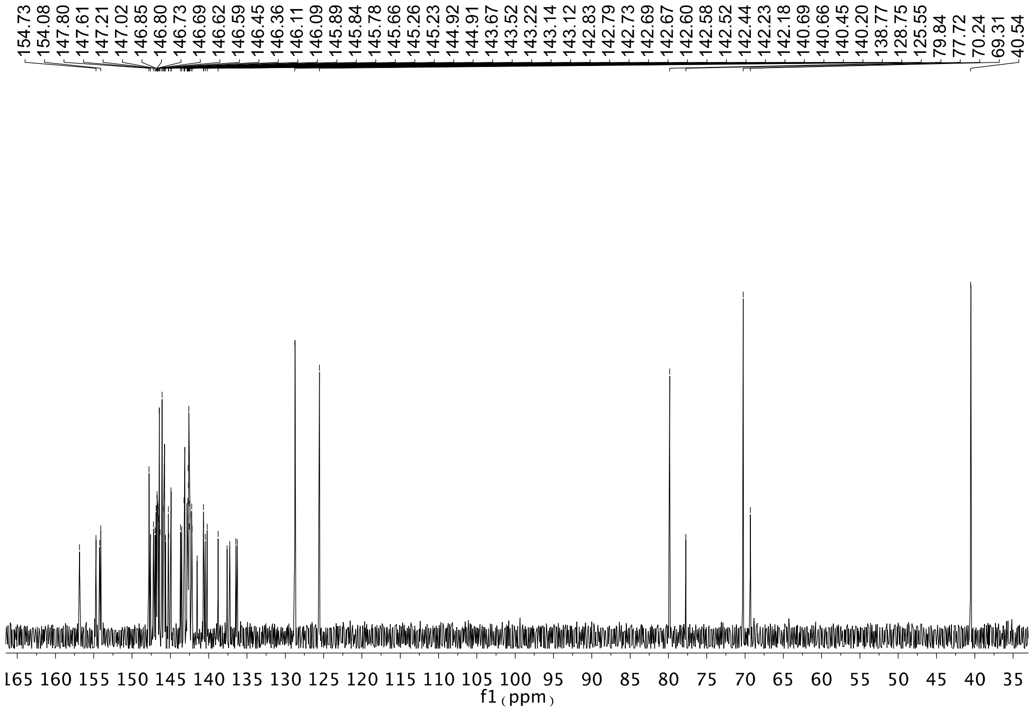

Figure S5. ^{13}C NMR spectrum of compound 60B in a mixture of CS_2 and acetone-d6**.**

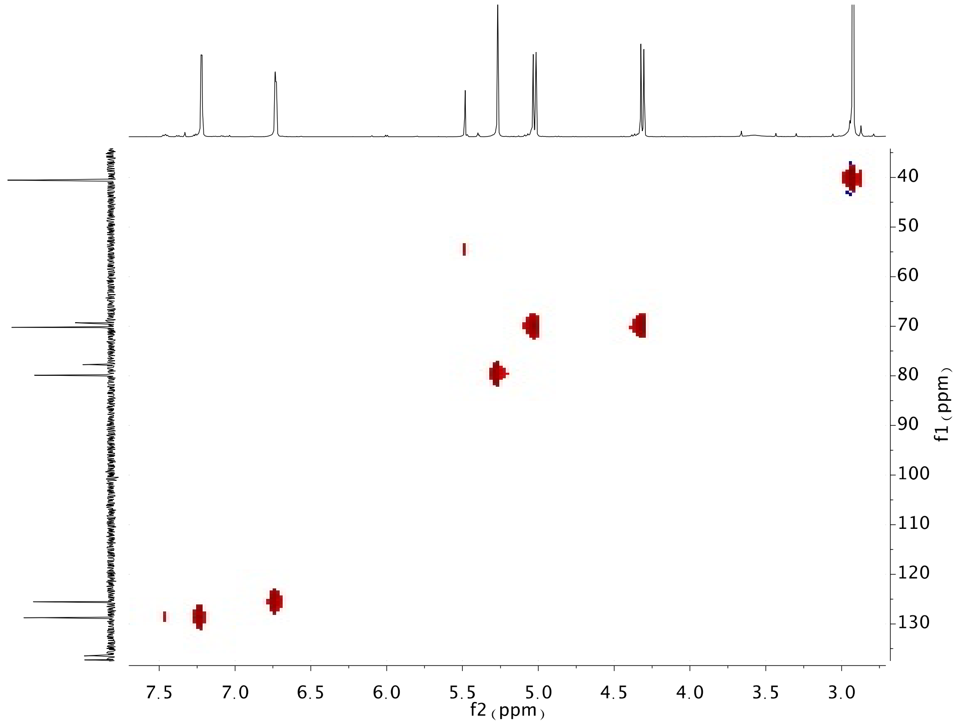

Figure S6. HSQC spectrum of compound 60B in a mixture of CS_2 and acetone-d6.

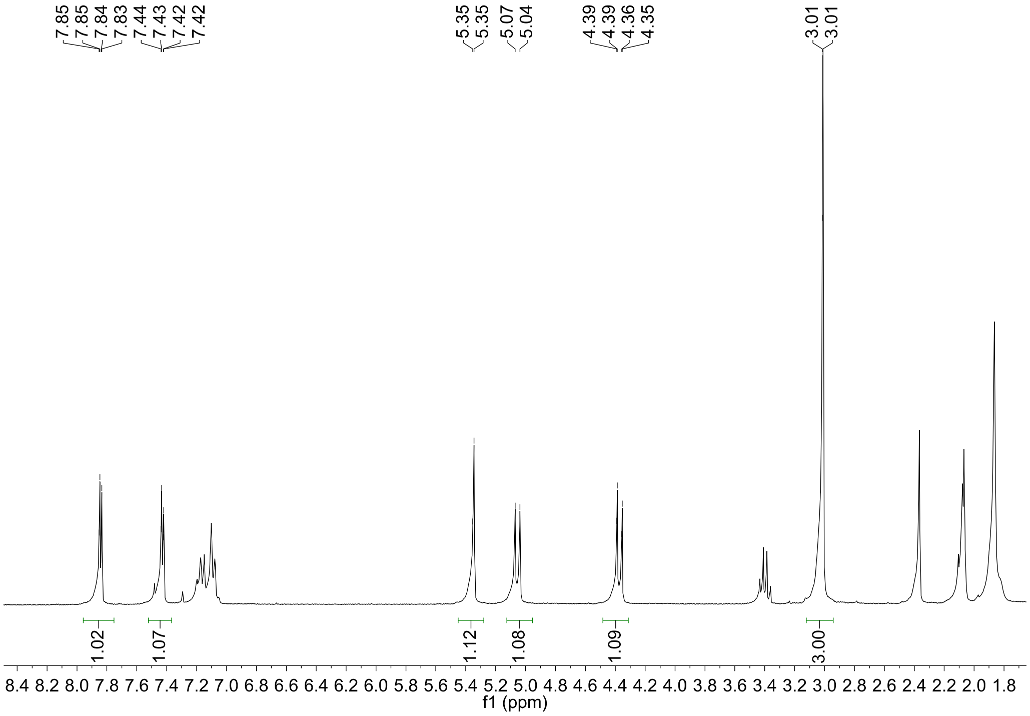

Figure S7. ^{1}H NMR spectrum of compound 60C in a mixture of CS_2 and acetone-d6.

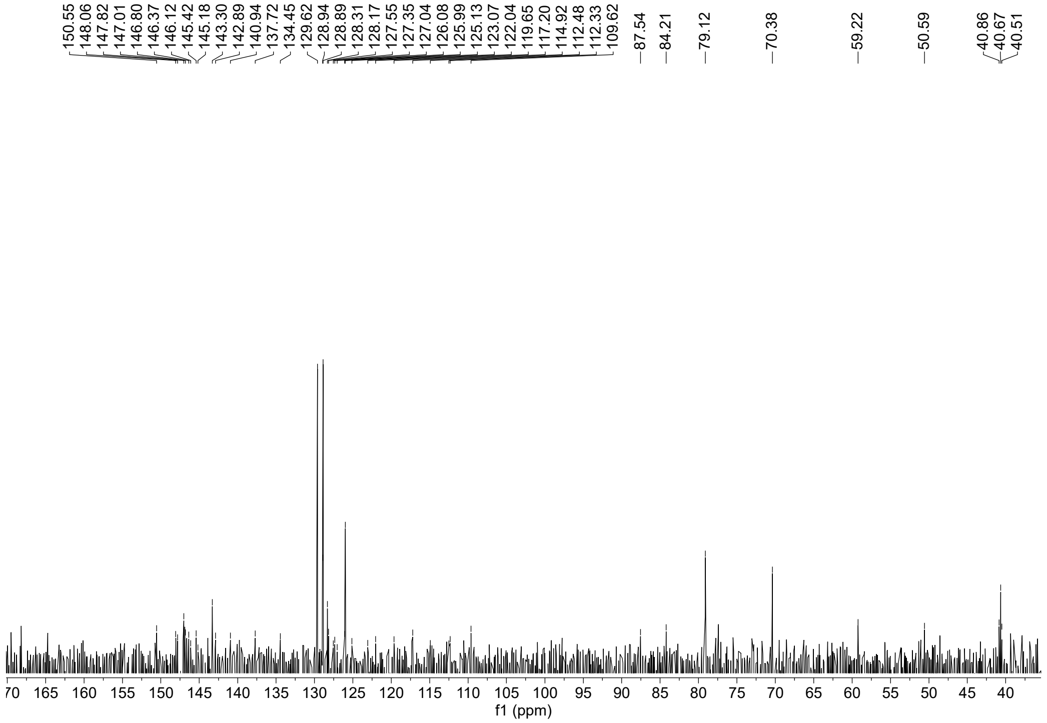

Figure S8. ^{13}C NMR spectrum of compound 60C in a mixture of CS_2 and acetone-d6.

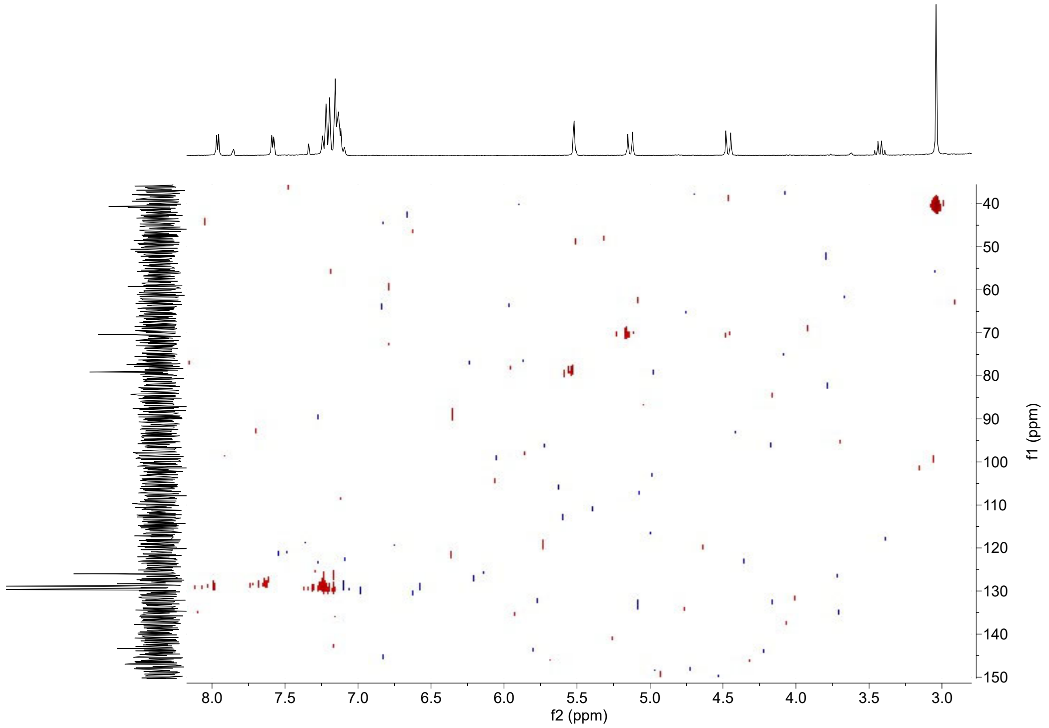

Figure S9. HSQC spectrum of compound 60C in a mixture of CS_2 and acetone-d6.

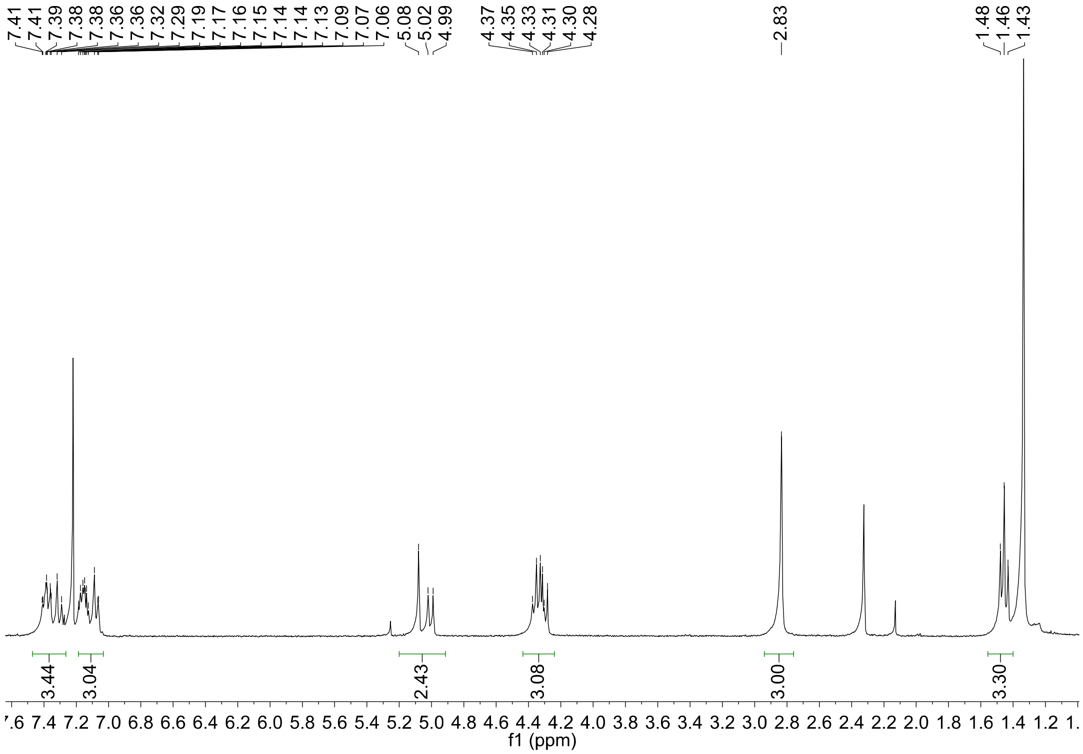

Figure S10. ^{1}H NMR spectrum of compound 60D in a mixture of CS_2 and chloroform-d.

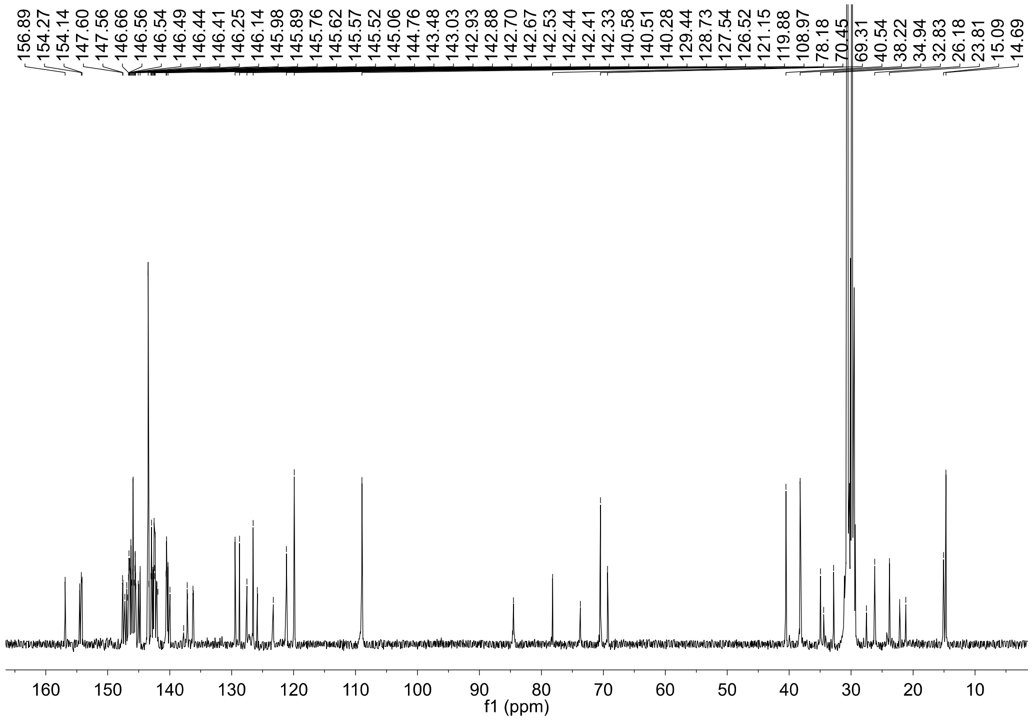

Figure S11. ^{13}C NMR spectrum of compound 60D in a mixture of CS_2 and chloroform-d.

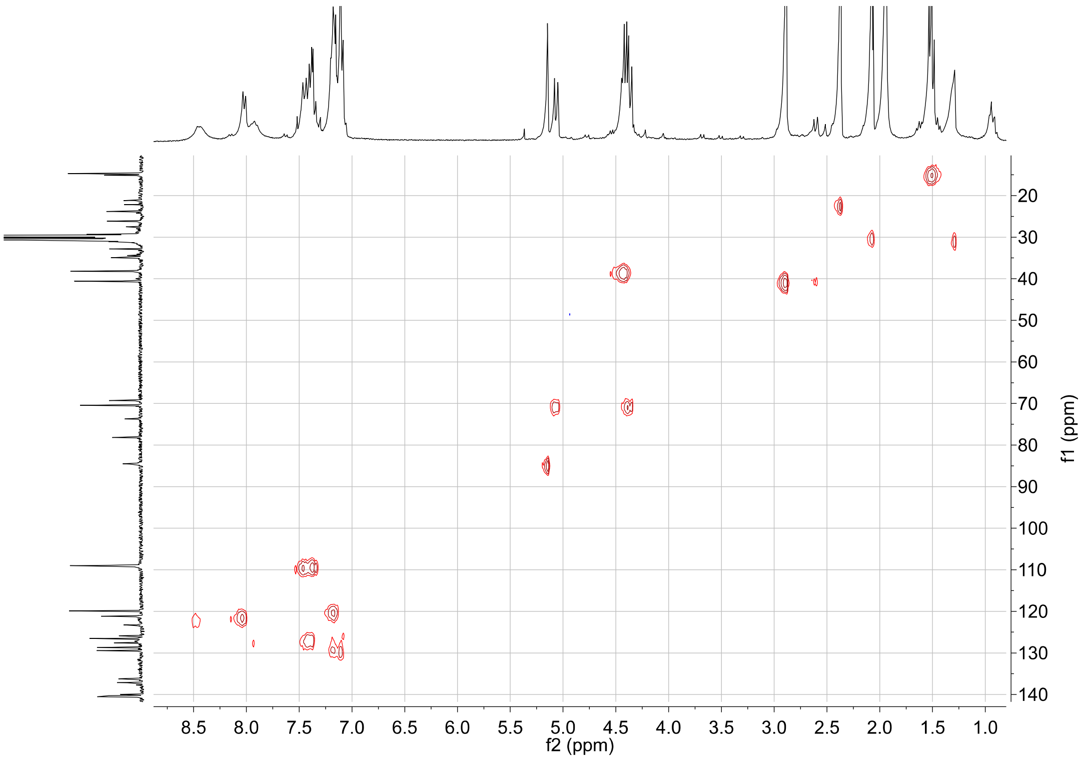

Figure S12. HSQC spectrum of compound 60D in a mixture of CS_2 and chloroform-d.

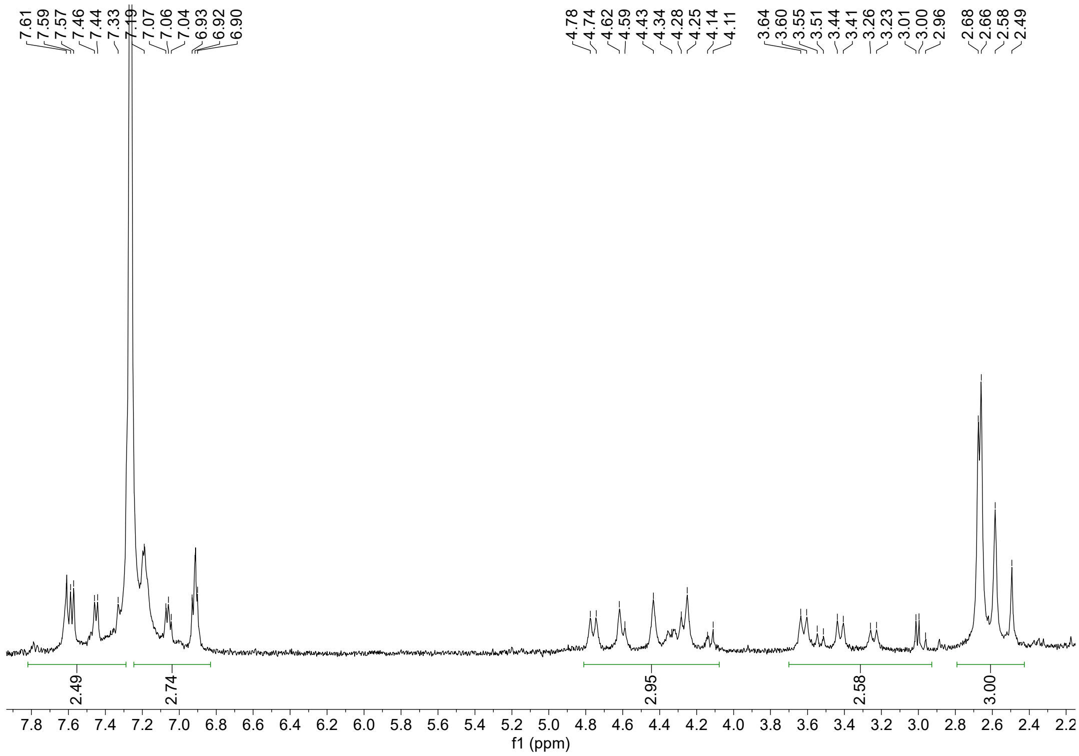

Figure S13. ^{1}H NMR spectrum of compound 70A in Choroform-d.

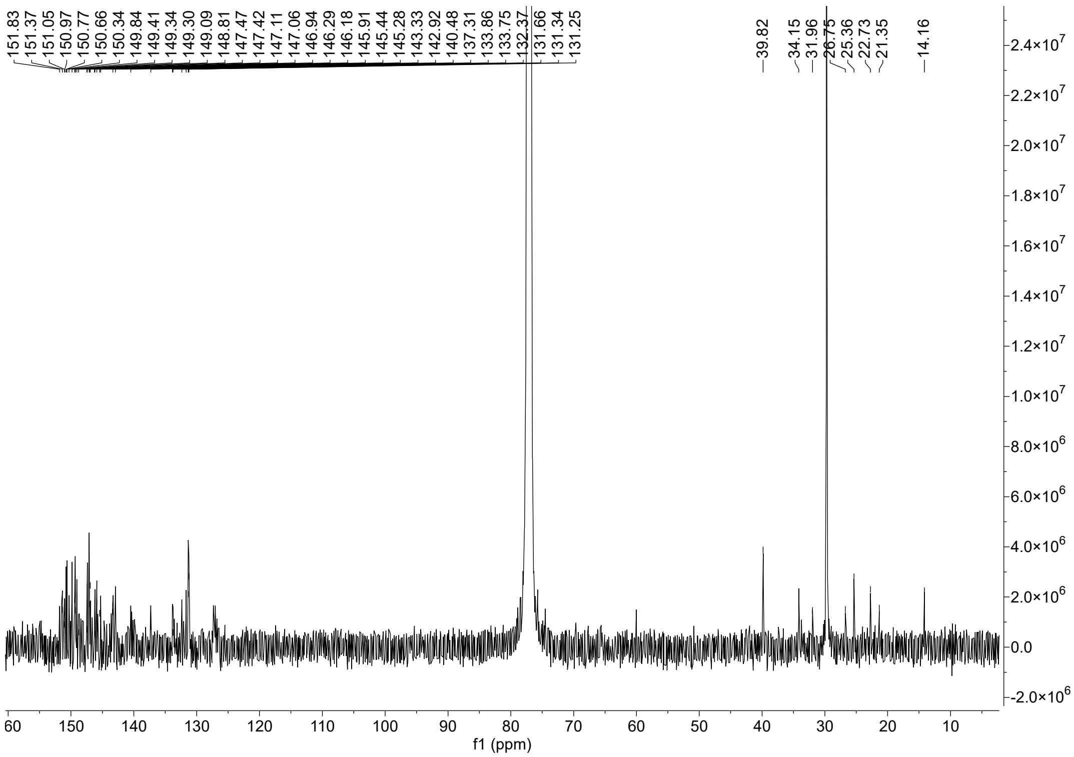

Figure 14. ^{13}C NMR spectrum of compound 70A in Choroform-d.

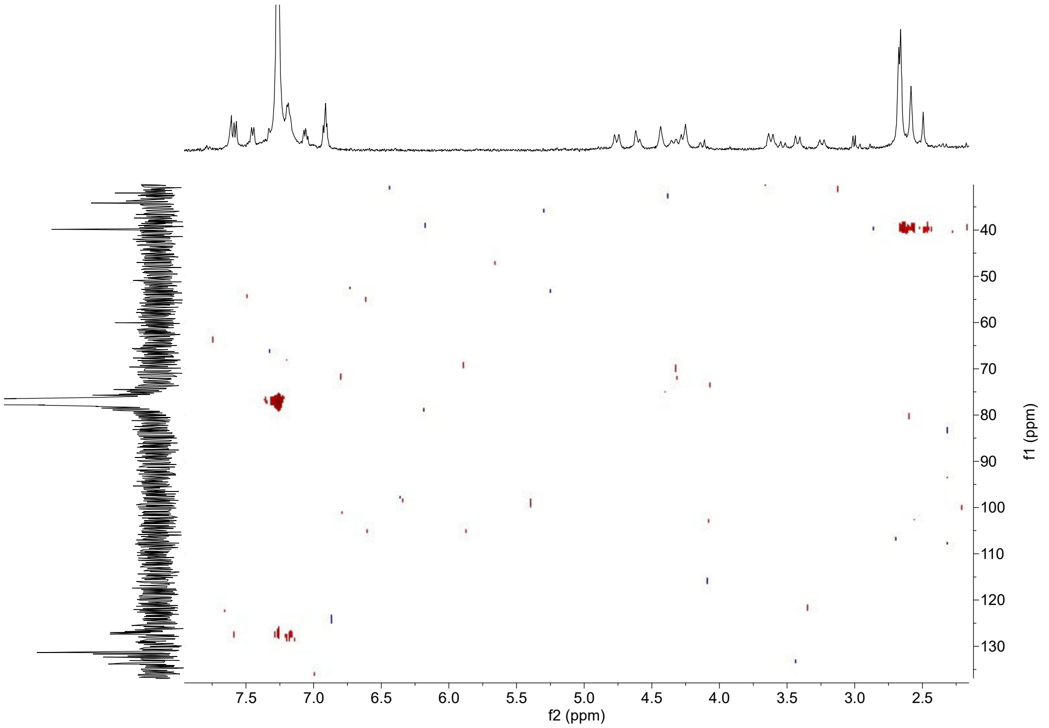

Figure S15. HSQC spectrum of compound 70A in Choroform-d.

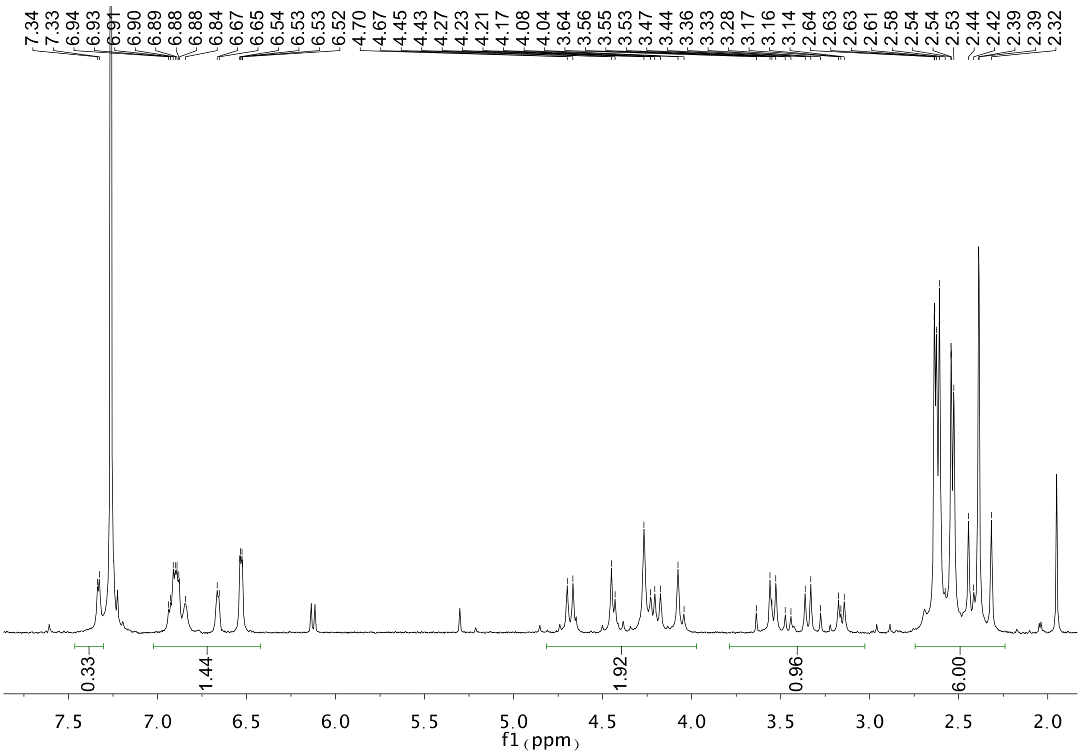

Figure S16. ^{1}H NMR spectrum of compound 70B in chloroform-d.

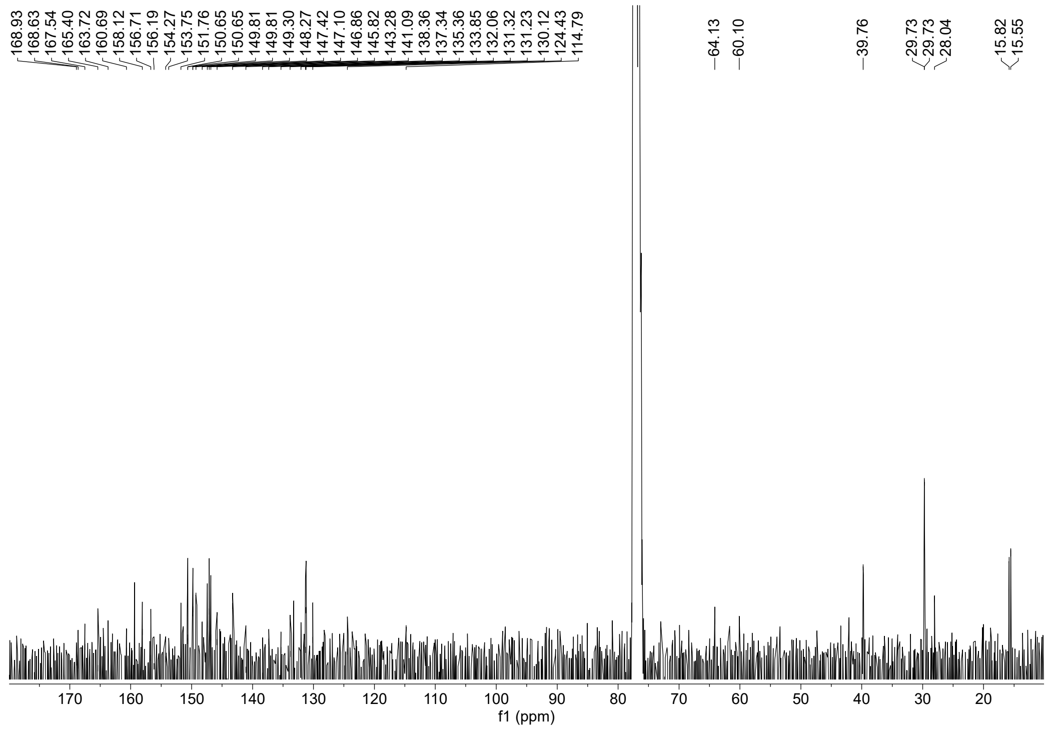

Figure S17. ^{13}C NMR spectrum of compound 70B in chloroform-d.

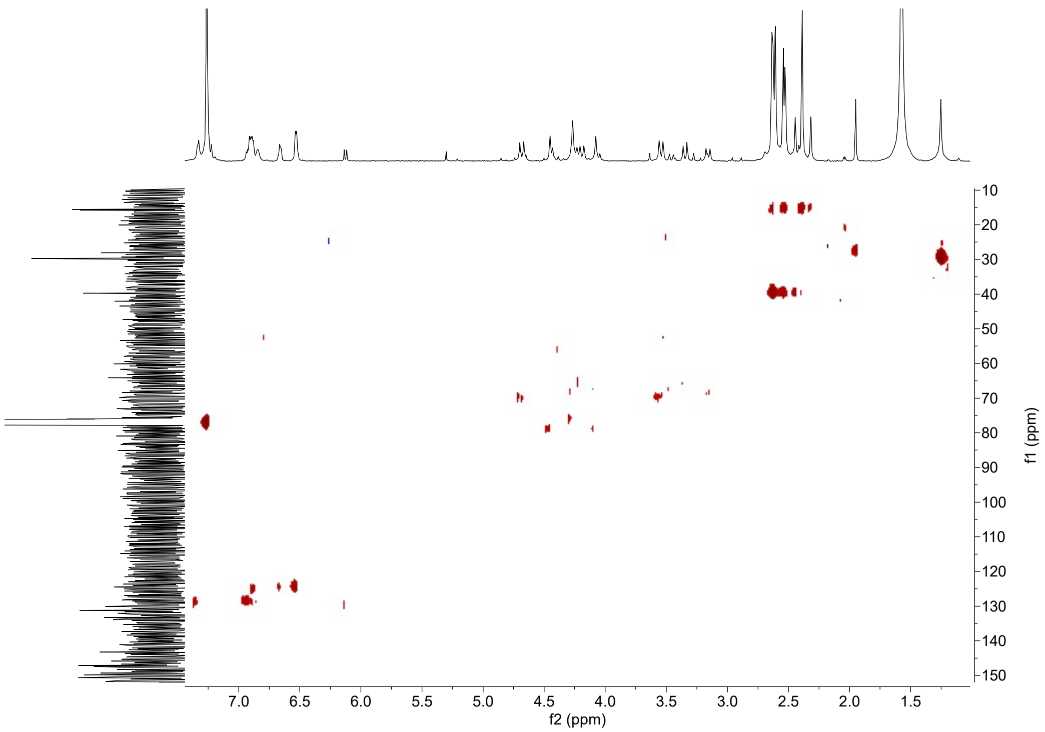

Figure S18. HSQC spectrum of compound 70B in chloroform-d.

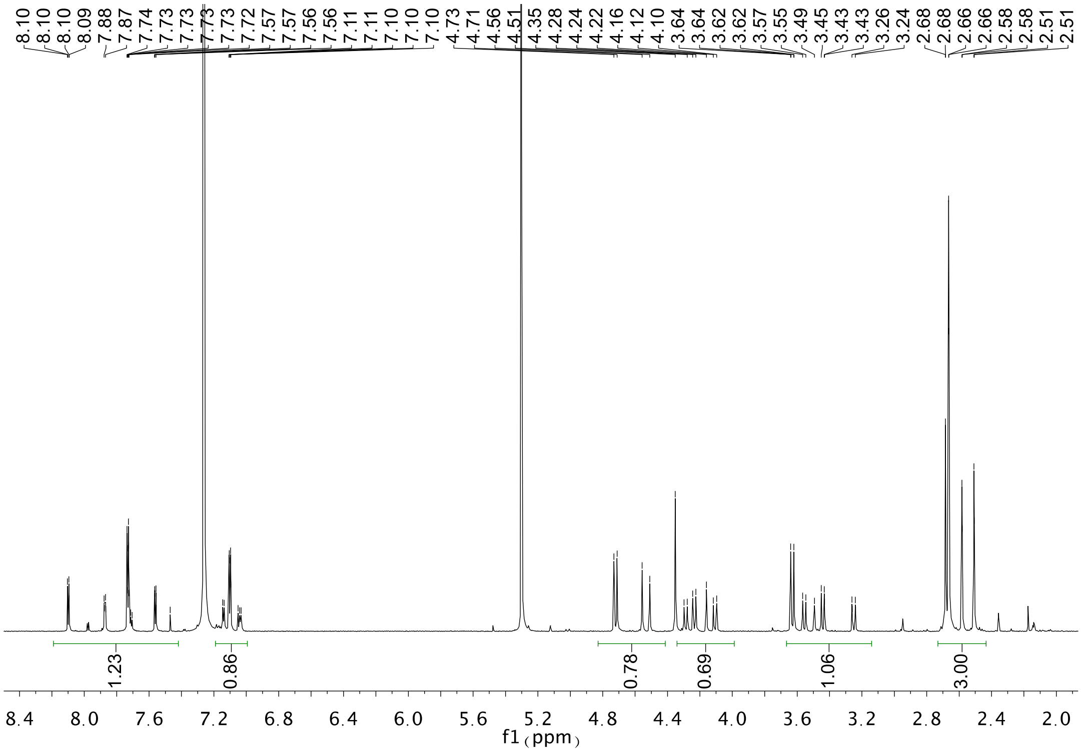

Figure S19. ^{1}H NMR spectrum of compound 70C in a mixture of CS_2 and chloroform-d.

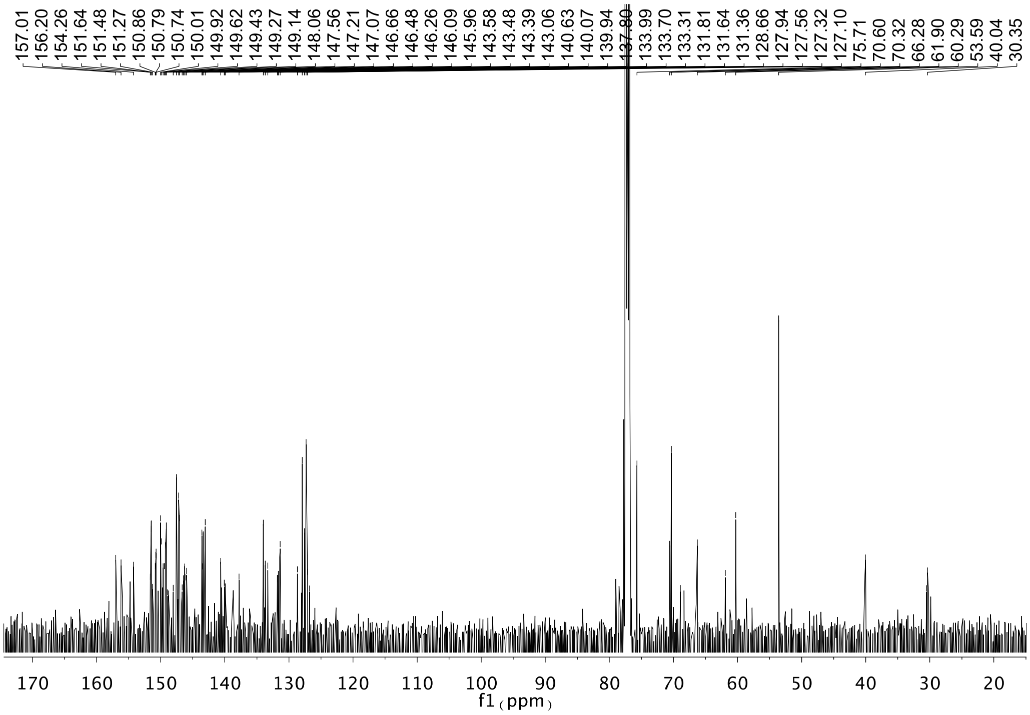

Figure S20. ^{13}C NMR spectrum of compound 70C in a mixture of CS_2 and chloroform-d.

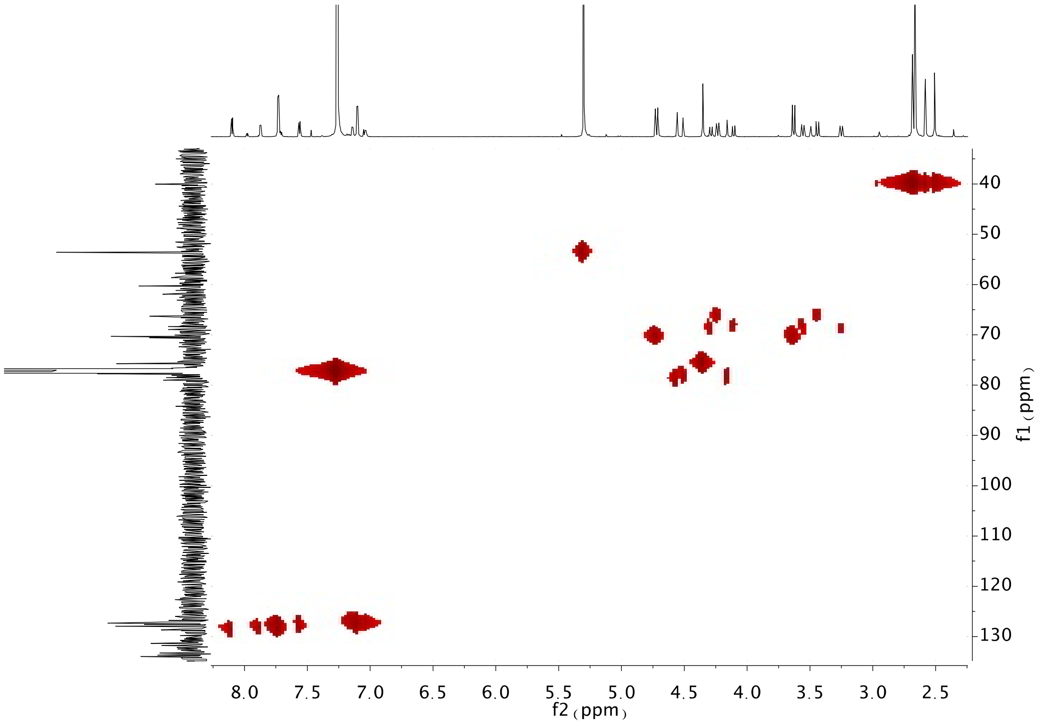

Figure S21. HSQC spectrum of compound 70C in a mixture of CS_2 and chloroform-d.

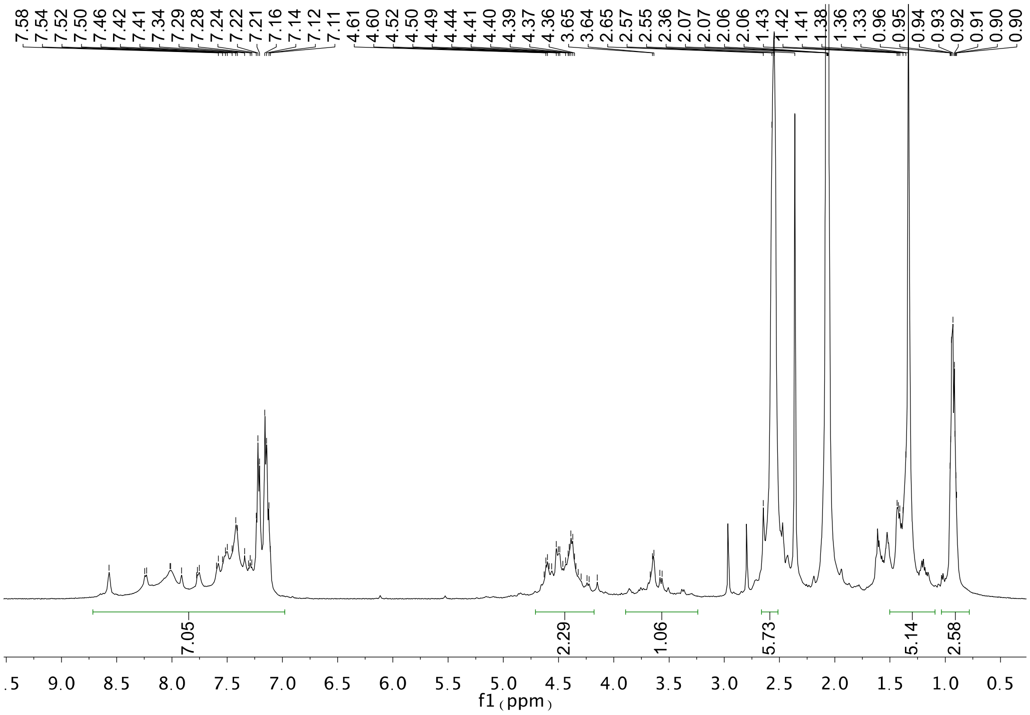

Figure S22. ^{1}H NMR spectrum of compound 70D in a mixture of CS_2 and acetone-d6.

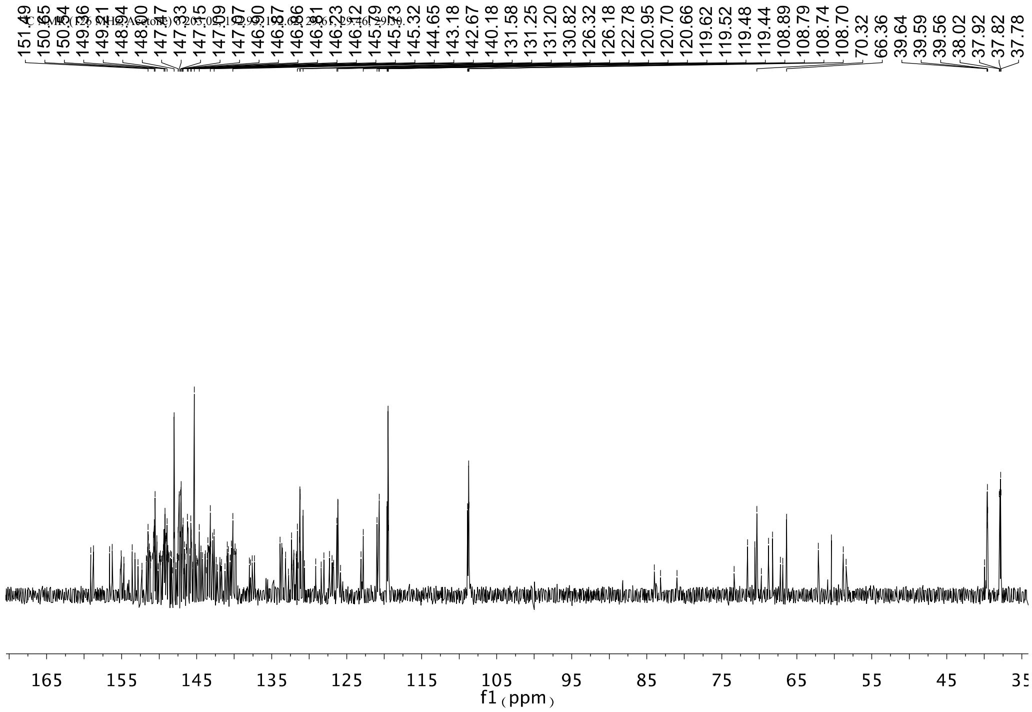

Figure S23. ^{13}C NMR spectrum of compound 70D in a mixture of CS_2 and acetone-d6.

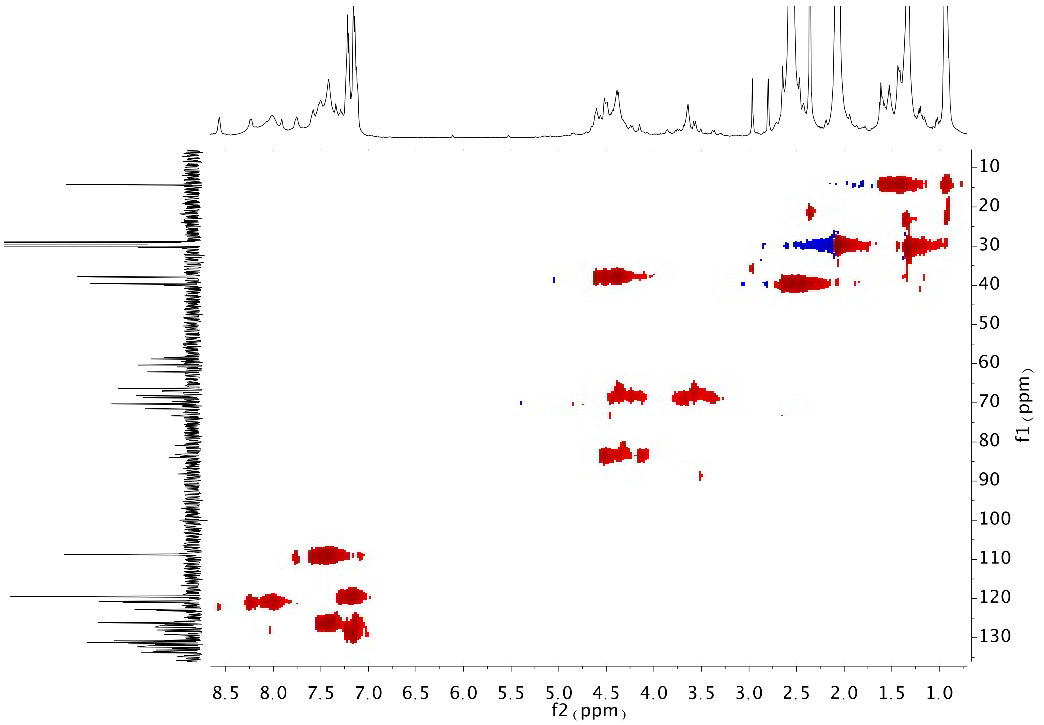

Figure S24. HSQC spectrum of compound 70D in a mixture of CS_2 and acetone-d6.

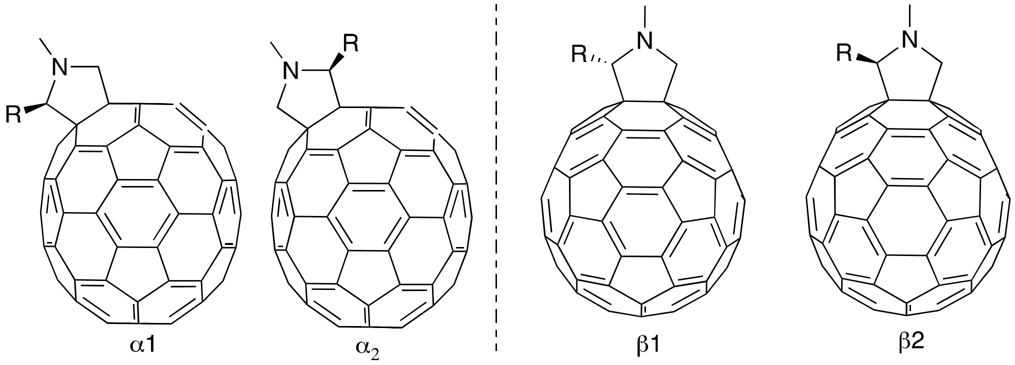

Figure S25. Structure of the four different C_{70}-fulleropyrrolidine isomers.

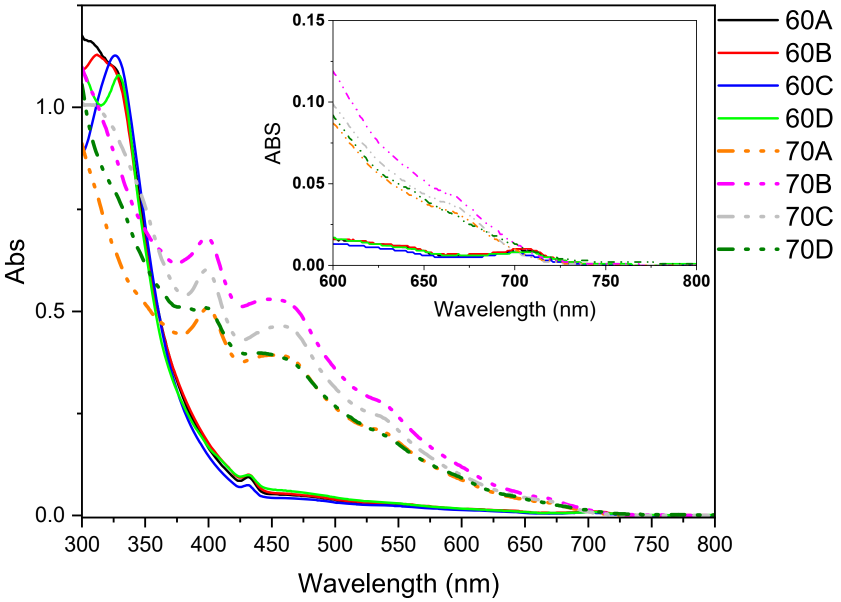

Figure S26. UV-Vis absorption spectra of the pure fullerenes in 1,2-dichlorobenzene.

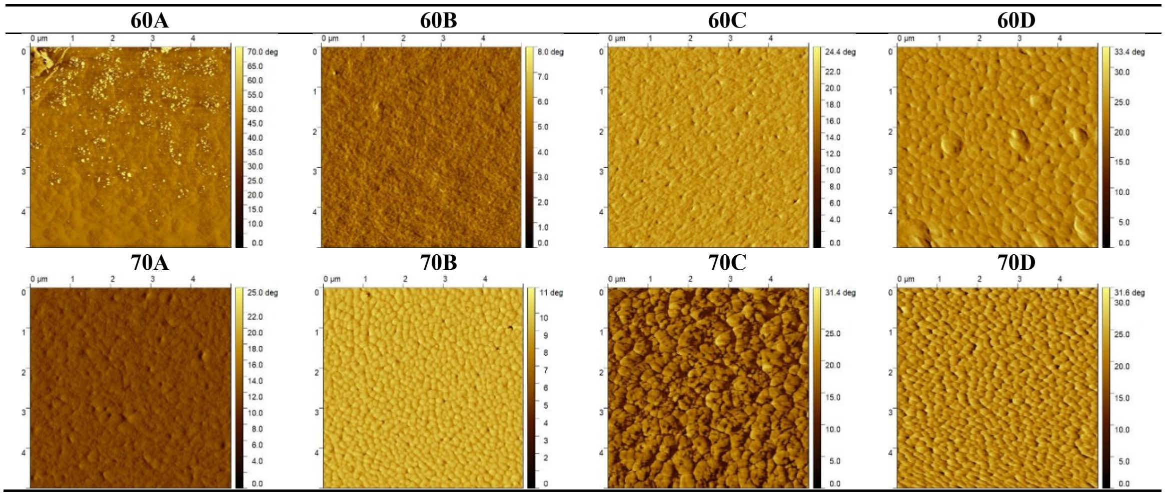

Figure S27. AFM phase images of PffBT4T-2OD based bulk-heterojunction films with different fullerenes.
